# Three-dimensional voltage imaging in live larval zebrafish brains using fully genetically encoded voltage indicator

**DOI:** 10.1038/s41598-026-58107-8

**Published:** 2026-06-16

**Authors:** Eun-Seo Cho, Minho Eom, Shihao Zhou, Gyuri Kim, Soi Kim, Cheol-Hee Kim, Kiryl D. Piatkevich, Young-Gyu Yoon

**Affiliations:** 1 School of Electrical Engineering, KAIST, Daejeon, Republic of Korea; 2 School of Life Sciences, Westlake University, Hangzhou, Zhejiang China; 3 Westlake Laboratory of Life Sciences and Biomedicine, Hangzhou, Zhejiang China; 4 Institute of Basic Medical Sciences, Westlake Institute for Advanced Study, Hangzhou, Zhejiang China; 5 Department of Biology, Chungnam National University, Daejeon, Republic of Korea; 6 KAIST Institute for Human Augmentation Convergence, Daejeon, Republic of Korea; 7 Department of Semiconductor System Engineering, KAIST, Daejeon, Republic of Korea

**Keywords:** Biological techniques, Biotechnology, Engineering, Neuroscience

## Abstract

Voltage imaging has emerged as a powerful tool for recording membrane potential changes in living cells, offering a direct measurement of rapid neuronal events with high temporal precision. Since the brain is a three-dimensional circuit, it is essential to record signals across a volume. However, achieving effective three-dimensional voltage imaging over large neuronal populations remains challenging due to the need for high imaging speed, high signal-to-noise ratio, and extensive volume coverage. In this study, we demonstrate in vivo three-dimensional voltage imaging in larval zebrafish using oblique plane microscopy and QFDBD-QUAS-driven expression of the genetically encoded voltage indicator Ace-mNeon2-Kv2.1, achieving volumetric imaging rates of up to 200 volumes per second (VPS). This approach enables dye-free voltage imaging, simplifying experimental workflows and improving the reproducibility of in vivo voltage imaging experiments for investigating neuronal circuit dynamics in the living zebrafish animal model.

**Supplementary Information:**

The online version contains supplementary material available at https://doi.org/10.1038/s41598-026-58107-8.

## Introduction

Achieving high temporal precision in recording the activities of a large population of neurons is essential for investigating causality within neural circuits^1–5^, which is arguably a prerequisite for understanding complex neural interactions and unraveling the mechanisms underlying brain functions. Voltage imaging^1–22^ is a promising solution to this challenge owing to the massively parallel nature of the image acquisition process, as each pixel on the image sensor operates as a measuring site. The major challenge lies in simultaneously achieving spatial resolution, temporal resolution, signal-to-noise ratio (SNR), and field-of-view that are sufficient to detect action potentials from a large number of neurons.

Unfortunately, regardless of the microscopy method, these factors are fundamentally limited by the number of photons that can be obtained from the sample in a given period, which must be allocated across the four dimensions of space and time (*x*, *y*, *z*, *t*). Consequently, allocating more photons to one dimension reduces the photons available for others, resulting in inevitable competition among spatial resolution, imaging speed, and SNR. Therefore, identifying the optimal balance among these metrics is crucial to ensure that each one meets the necessary performance thresholds. For voltage imaging in intact brains, neuronal action potentials evolve on the millisecond timescale, demanding near kilohertz-class sampling rates. Each voxel (or plane) must be acquired with short exposure times, reducing collected photons and degrading SNR. These constraints have historically pushed brain-wide or fast three-dimensional (3-D) voltage imaging toward exceptionally bright indicator strategies and highly light-efficient optics.

Recent two-photon imaging methods^18,19^ have demonstrated voltage imaging of more than 100 neurons, and multiplane confocal microscopy^20^ has demonstrated volumetric voltage recordings in densely labeled neuronal populations. However, achieving sampling rates required for voltage imaging compromises the number of neurons distributed across brain volumes due to the sequential measurement scheme. Recording across distributed brain regions requires imaging methods that integrate fluorescence signals from many spatial locations in parallel rather than distributing the limited photon budget through serial scanning. To fully leverage the parallel data acquisition capabilities of CMOS image sensors, we must use camera-based microscopy methods^21–28^, such as light-sheet microscopy or light-field microscopy, for large-scale volumetric imaging.

To effectively implement an advanced imaging technique, we selected the zebrafish (*Danio rerio*) as our model organism. Larval zebrafish are ideal for in vivo imaging studies due to their optical transparency at the larval stage and the well-characterized nature of their nervous system^21–28^. This transparency, combined with genetically encoded voltage indicators (GEVIs)^6–10,14,15,17^, allows for non-invasive optical interrogation of neural activity and structure at the whole-brain level, making them an ideal testbed for advanced imaging techniques. Light-field microscopy has demonstrated its capability for whole-brain calcium imaging in the larval zebrafish by capturing volumetric information in a single camera snapshot, but suffers from limitations in spatial resolution because the finite camera pixels are used to encode both spatial and angular information, resulting in an inherent spatio-angular trade-off. Unlike calcium imaging, where fluorescent molecules are located in the cytosol or nuclei, voltage imaging requires higher spatial resolution because the fluorescent molecules are localized to membranes in close contact with adjacent neurons^5,10^. Considering these factors, light-sheet microscopy stands as a promising choice due to its optical sectioning capability and high spatial resolution, which are necessary for imaging densely packed neurons in the larval zebrafish brain^21,23,25^, making it more suitable than light-field imaging for voltage imaging in the larval zebrafish, despite the success of light-field microscopy in whole-brain calcium imaging.

On the other hand, recent demonstrations of larval zebrafish voltage imaging have relied on chemigenetic indicators^14,16,17,21,22^ that combine genetic targeting with synthetic fluorophores, enabling fast brain-wide recordings with Positron2^21^ and spinal cord recordings with Voltron2^22^ at high volumetric rates (e.g., 200–500 Hz). However, chemigenetic voltage imaging introduces experimental dependencies that are undesirable for routine, scalable, and reproducible whole-brain workflows. Both Positron2- and Voltron2-class indicators require exogenous HaloTag ligands (JF dyes), imposing additional handling steps (staining, washout, and timing constraints) and introducing sample-to-sample variability through dye delivery and clearance. For example, in larval zebrafish Positron2-Kv experiments, larvae were incubated in 3 µM JF525-HaloTag ligand for two hours and residual dye was washed off prior to screening and imaging; the authors further note the need to minimize the gap between staining and imaging to reduce unlabeled neurons born post-staining^21^. Similarly, Voltron2 protocols require in vivo dye incubation in zebrafish (e.g., JF552-HaloTag ligand), creating an additional experimental axis that can affect background fluorescence and effective SNR. Beyond workflow complexity, reliance on synthetic dyes increases per-experiment cost and raises the possibility of unintended physiological effects from dye exposure and/or incomplete clearance, which is particularly problematic for longitudinal or high-throughput experiments.

In this study, we optimized a single-objective light-sheet microscopy known as oblique plane microscopy for volumetric imaging of neuronal voltage across extensive regions of the brain and spinal cord, achieving imaging rates of up to 200 volumes per second (VPS). Our approach enables dye-free three-dimensional voltage imaging of neuronal populations in the live larval zebrafish brain. We utilized the QFDBD–QUAS transgenesis based fully genetically encoded voltage indicator Ace-mNeon2-Kv2.1 to image zebrafish larvae, simplifying the experimental procedures compared to chemigenetic voltage indicators such as Voltron, which require additional dye loading and rinsing steps.

## Results

### Implementation of oblique plane microscope

**Fig. 1:**
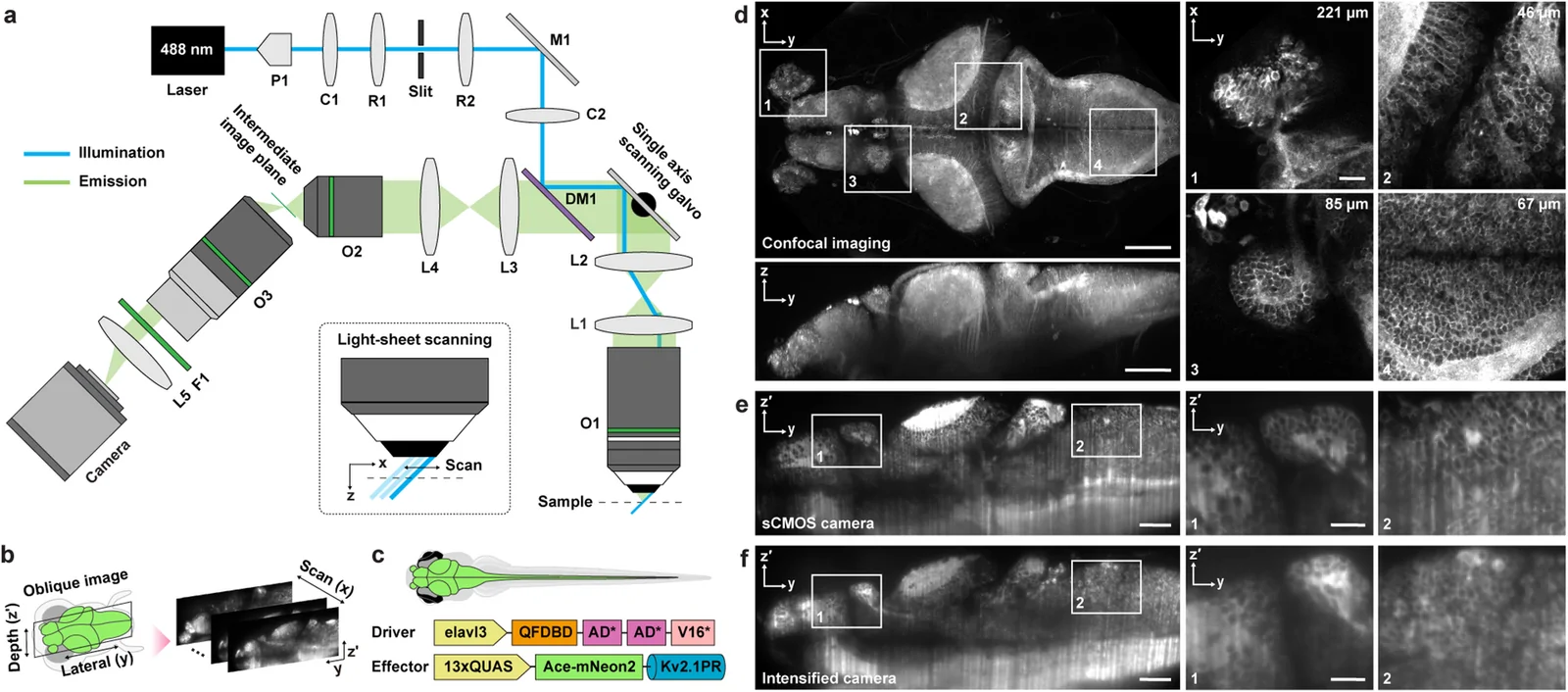
Oblique plane microscopy and fully genetically encoded voltage indicator for three-dimensional voltage imaging. (**a**) Schematic diagram of the oblique plane microscope (OPM). The emission path includes the primary objective (O1), secondary objective (O2), third objective lens (O3), series of achromatic lenses (L1 to L4), tube lens (L5), and emission filter (F1). The illumination path consists of Powell lens (P1), plano-convex cylindrical lenses (C1 and C2), achromatic relay lenses (R1 and R2), right-angle mirror (M1), and dichroic mirror (DM1). The position of the oblique light-sheet across the sample was adjusted along the scanning direction (*x*-axis) using a galvo mirror (dashed box). (**b**) Volumetric imaging with oblique plane microscopy. Sequentially acquired oblique images (*yz*′ planes) are stacked along the scanning direction (*x*-axis) to reconstruct three-dimensional volumes. (**c**) Illustration of transgenic larval zebrafish expressing Ace-mNeon2-Kv2.1 under the QFDBD–QUAS genetic system. (**d**) Volumetric structural imaging of the larval zebrafish brain using a point-scanning confocal microscope. Magnified views of the white boxed regions (1–4) are shown on the right at selected depths, corresponding to the olfactory epithelium (1), optic tectum (2), habenula (3), and medulla oblongata (4). Scale bars, 100 μm (left) and 20 μm (right). (**e**) Average projections of single-plane images acquired with OPM at 200 Hz using an sCMOS camera. Magnified views of the boxed regions (1–2) are shown on the right. Scale bars, 50 μm (left) and 20 μm (right). (**f**) Average projections of single-plane images acquired with OPM at 200 Hz using an intensified camera. Magnified views of the boxed regions (1–2) are shown on the right. Scale bars, 50 μm (left) and 20 μm (right).

We implemented an oblique plane microscope (OPM)^29–35^ optimized for volumetric imaging of neuronal voltage across extensive brain regions. The detection module was designed to be interchangeable, allowing operation with either a scientific CMOS (sCMOS) camera or a high-speed intensified camera^32^. The schematic of the OPM illustrated the emission and illumination paths (Fig. 1a). Our OPM system was capable of volumetric imaging over a volume of 127 μm (*x*) × 678 μm (*y*) × 217 μm (*z*) at 200 VPS. To simultaneously achieve the speed, resolution, and volumetric field-of-view requirements, we performed simulations using a commercial optical simulation software to determine the optimal optical configurations for our single-objective light-sheet microscope. These simulations focused on minimizing optical aberrations within the target volumetric field of view (Supplementary Fig. 1). By carefully adjusting the lens combinations, we achieved a sufficient field of view for three-dimensional imaging of the larval zebrafish brain with low optical aberrations.

In OPM, a primary objective (O1) was used to simultaneously generate an oblique light-sheet in the sample space and detect the emitted fluorescence light. For excitation, we used a 488 nm laser and a Powell lens (P1) to generate a uniform light-sheet. The fluorescence emitted from the sample at O1 is relayed to the intermediate image plane at the secondary objective (O2) using a series of achromatic lenses (L1 to L4), which conjugate the pupil planes of O1 and O2. We employed the structure of a Plössl lens for L2 and L3 to reduce optical aberrations. The magnification (1.33) of the intermediate image corresponds to the refractive index (n) ratio between the media of O1 (water, *n* = 1.33) and O2 (air, *n* = 1.00). The intermediate image was focused through a third objective lens (O3) and a camera tube lens (L5) onto an intensified camera after passing through an emission filter. The effective pixel size of the camera at the sample space was 0.53 μm, which increased to 1.06 μm after applying 2 × 2 binning. The detection module could also be configured with the sCMOS camera paired with a different camera tube lens (L5), resulting in an effective pixel size of 0.98 μm after 2 × 2 binning. In both configurations, 2 × 2 binning was used to increase acquisition speed by reducing the number of pixels read out per frame.

For volumetric imaging, the position of the oblique light-sheet across the sample was adjusted along the scanning direction (*x*-axis) using a galvo mirror, while the intermediate oblique images (*yz*’ plane images) of the oblique light-sheet remained stationary (Fig. 1a). The volumetric image was then constructed by stacking these sequential oblique images (Fig. 1b). This process includes using an affine transformation to adjust for the oblique light-sheet angle^31–33^. The scanning galvo mirror was controlled using a sawtooth function at the desired volume rate, which was an analog signal from a DAQ board. The DAQ board was synchronized with the trigger output signals from the intensified camera. The volumetric imaging rate was determined by the frame rate of the camera and the number of oblique images in a single volume. The distance between adjacent *yz*’ plane images, which corresponded to the step size along the *x*-axis, was determined based on the volume scan rate and the target volumetric field of view. For instance, when imaging specimens at 200 VPS, the camera operated at a frame rate of 5,600 Hz and recorded 28 images sequentially in each period of the galvo driving signal, including discarded images due to the settling time of the galvo scanner. For image acquisition, we utilized commercial image acquisition software for the intensified camera and custom software implemented in MATLAB to control the galvo and DAQ board. We characterized the spatial resolution of the volumetric imaging setup by measuring the point spread function (PSF) using 200 nm diameter fluorescent beads, with a full width at half maximum (FWHM) extracted via Gaussian fitting. The FWHM values along the x, y, and z axes were approximately 2.10 μm, 1.80 μm, and 2.40 μm, respectively (Supplementary Fig. 2).

### Fully genetically encoded voltage indicator expression based on QFDBD–QUAS transgenesis

For the fully genetically encoded voltage indicator, we selected Ace-mNeon2-Kv2.1, a bright rhodopsin-based GEVI with fast voltage-dependent fluorescence responses and Kv2.1-mediated soma-enriched membrane localization^36^. These properties made Ace-mNeon2-Kv2.1 well suited for high-speed light-sheet voltage imaging because short exposure times in volumetric acquisition impose a stringent photon-budget constraint, while soma-enriched membrane localization facilitates cellular-resolution segmentation and voltage signal extraction from densely labeled neuronal tissue. This choice shifted the burden of SNR optimization from dye chemistry to genetic control of indicator abundance at the membrane. In this framework, two requirements became central: (i) achieving sufficiently high expression to support photon-limited volumetric readout, and (ii) enabling tunable expression to avoid excessive overexpression, which has been noted as a potential source of electrophysiological perturbation (e.g., membrane loading effects) in the broader GEVI literature.

Binary expression systems provide a direct route to controlling expression strength and specificity in zebrafish. The Gal4-UAS system^37^ is widely used, but UAS arrays are short tandem repeats that are susceptible to CpG methylation-associated transcriptional silencing and variegation across generations, producing mosaic or weakened expression in stable lines^38^, an outcome that is particularly detrimental for brain-wide voltage imaging where uniform labeling is essential. The Neurospora-derived QF-QUAS system provides an alternative binary expression strategy in which QF-dependent reporter activation can be separated from the promoter driving the transactivator^39^. However, broad expression of full-length QF or QF2 can cause severe developmental toxicity in zebrafish, motivating the development of optimized QF-based systems that retain QUAS-dependent reporter activation while reducing toxicity^40^.

Several QF-based strategies have been developed to address this toxicity issue in zebrafish. One approach used chimeric or attenuated QF variants, including QFGal4, which retains the QF DNA-binding domain but replaces the native QF activation module with the Gal4 activation domain, thereby supporting QUAS reporter activation with reduced developmental toxicity^40^. Another approach, the IQ-Switch system, similarly separates QF DNA binding from the native QF activation domain and uses engineered activation modules to generate QFDBD-based drivers^41^. In this study, we adopted an IQ-Switch-derived QF variant, QFDBD-2xAD*-VP16*. In the IQ-Switch framework, removal of the inducible module yields a constitutively active “EQ-On” driver that provides strong activation with minimal toxicity, and importantly supports stepwise tuning of expression by varying the number of QUAS repeats (with the strongest reporter signals observed at 13xQUAS). Moreover, IQ-Switch analysis supports methylation-independent QUAS activation, directly addressing the epigenetic vulnerability that often underlies UAS mosaicism in zebrafish.

Based on these considerations, we generated and validated a dye-free, brain-wide voltage imaging transgenic strategy using the nearly pan-neuronal HuC/elavl3 promoter to drive QFDBD-2xAD*-VP16* expression and a new transgenic line *Tg(13xQUAS: Ace-mNeon2-Kv2.1)*, as shown in Fig. 1c. The HuC/elavl3 promoter was chosen because it has been widely used to label differentiated neurons throughout the zebrafish nervous system, enabling broad neuronal expression suitable for brain-wide imaging^42^. The resulting QFDBD-QUAS transgenic larval zebrafish showed robust pan-neuronal expression across the brain, with bright fluorescence suitable for photon-limited volumetric acquisition. The ability to combine a high-repeat QUAS effector (13xQUAS) with a low-toxicity QFDBD driver provided a practical expression-tuning axis to achieve high brightness without requiring exogenous dyes. Collectively, these results establish QFDBD-QUAS-driven Ace-mNeon2-Kv2.1 as a fully genetically encoded route to larval zebrafish 3-D voltage imaging, removing dye-handling steps while retaining the SNR advantages necessary for high-speed volumetric microscopy. We imaged the larval zebrafish brain at 6 days post-fertilization (dpf) using point-scanning confocal microscopy (Fig. 1d, Supplementary Video 1). Fluorescence was detected in neuronal structures across the brain (Fig. 1d). In volumetric structural imaging of the larval zebrafish brain, Ace-mNeon2-Kv2.1 appeared to be more soma localized than Ace-mNeon2 (Supplementary Fig. 3).

We next assessed whether neuronal structures remained distinguishable under the high-speed light-sheet imaging conditions used for voltage recordings. Single-plane images acquired at 200 Hz showed neuronal cell bodies with visible membrane contrast using both a sCMOS camera (Fig. 1e) and an intensified camera (Fig. 1f). Representative planes extracted from volumetric recordings at 200 VPS also maintained distinguishable neuronal structures. These results indicated that the expression level and optical configuration together provided sufficient structural visibility for volumetric voltage imaging (Supplementary Video 2).

### In vivo three-dimensional voltage imaging in larval zebrafish brain and spinal cord

**Fig. 2:**
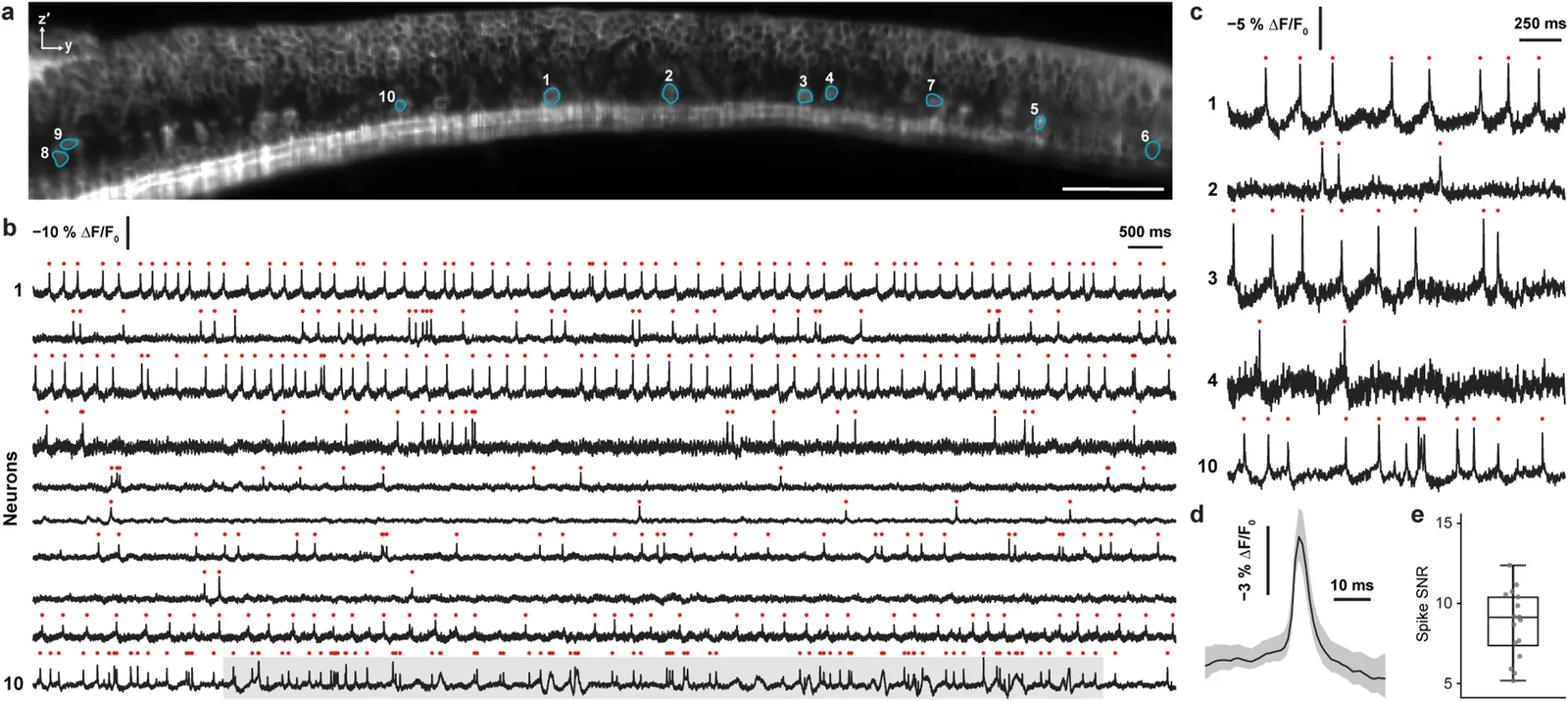
In vivo two-dimensional voltage imaging in the larval zebrafish spinal cord. (**a**) Single-plane voltage imaging of the spinal cord acquired at 1002 Hz. Manually segmented neuronal regions of interest (ROIs) are overlaid in blue. Scale bar, 50 μm. (**b**) Voltage traces extracted from the denoised imaging data using the neuronal ROIs marked in (**a**). Red dots indicate detected spike events. The gray-shaded area indicates the time period during which stripe artifacts were present in the recordings. (**c**) Expanded views of representative voltage transients from selected neurons. Denoised traces correspond to the first 3 s of the recordings in (**b**). Red dots indicate detected spike events. (**d**) Representative spike waveform obtained by averaging detected voltage spikes (mean ± s.d.). The solid line indicates the mean, and the shaded region represents ± s.d. The full width at half maximum (FWHM) is approximately 6 ms. (**e**) Spike signal-to-noise ratio (SNR) across segmented neurons (17 ROIs). Box-and-whisker plot with individual values overlaid.

We first recorded neuronal voltage changes in the larval zebrafish spinal cord at 1002 Hz (Fig. 2a). To minimize motion artifacts, the larval zebrafish were paralyzed during recordings. Thereafter, we applied a deep learning-based denoising technique, SUPPORT^43^, to suppress shot and Gaussian noise in the imaging data. Following denoising, individual neuronal cell bodies were more distinguishable, with the signals more apparent in the extracted voltage traces (Supplementary Fig. 4). Regions of interest (ROIs) corresponding to neuronal structures were manually segmented from the denoised imaging data due to limited automated detection and separation in densely labeled regions with overlapping neuronal structures (Supplementary Fig. 4). We observed spike-like voltage transients from spinal neurons (Fig. 2b,c and Supplementary Video 3). We note that stripe artifacts^21,44^, which arise from absorption or scattering of the excitation light-sheet by stationary pigmented structures or moving objects such as circulating blood cells and appear as shadows along the illumination direction, were present in both brain and spinal cord recordings, potentially obscuring neuronal signals and introducing time-varying intensity fluctuations on extracted voltage traces in affected frames (Fig. 2b and Supplementary Fig. 5). To characterize the temporal kinetics of these events, detected spikes from representative neurons were temporally aligned and averaged (Fig. 2d). The averaged voltage transient exhibited a full width at half maximum (FWHM) of approximately 6 ms. Spike SNR was quantified across segmented neurons (Fig. 2e). The measured FWHM provided a reference for determining the volumetric imaging rate required to capture rapid membrane potential dynamics. We therefore performed volumetric voltage imaging at 200 volumes per second, corresponding to a 5 ms sampling interval per volume.

**Fig. 3:**
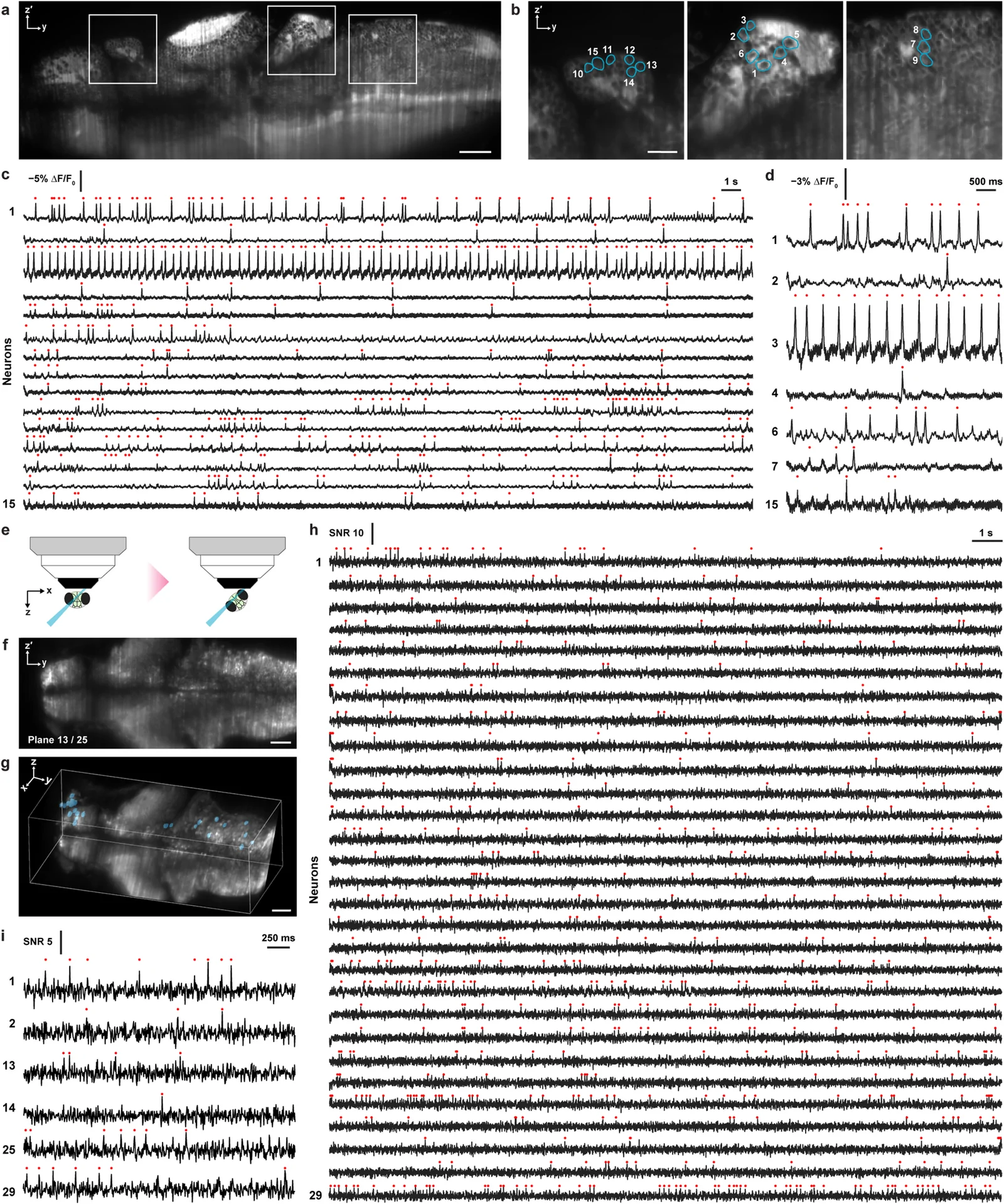
In vivo voltage imaging of neuronal activity in the larval zebrafish brain. (**a**) Single-plane voltage imaging of the larval zebrafish brain acquired at 200 Hz. Scale bar, 50 μm. (**b**) Magnified views of the boxed regions in (**a**). Manually segmented neuronal regions of interest (ROIs) are shown in the habenula, cerebellum, and medulla oblongata (from left to right). Scale bars, 20 μm. (**c**) Voltage traces extracted from the denoised imaging data using the neuronal ROIs marked in (**b**). Red dots indicate detected spike events. (**d**) Expanded views of representative voltage transients from selected neurons. Denoised traces correspond to the first 5 s of the recordings in (**c**). Red dots indicate detected spike events. (**e**) Illustration of the sample mounting strategy. Conventional mounting (left) and tilted mounting aligned with the oblique light-sheet (right). (**f**) Representative planes extracted from the oblique image stack acquired under tilted mounting. Scale bar, 50 μm. (**g**) Three-dimensional rendering of the reconstructed volumetric dataset. Segmented neurons are overlaid in blue. Scale bar, 50 μm. (**h**) Voltage traces extracted from the denoised imaging data using the neuronal ROIs across multiple brain regions marked in (**g**). Red dots indicate detected spike events. (**i**) Expanded views of representative voltage transients from selected neurons. Denoised traces correspond to the first 5 s of the recordings in (**h**). Red dots indicate detected spike events.

**Fig. 4:**
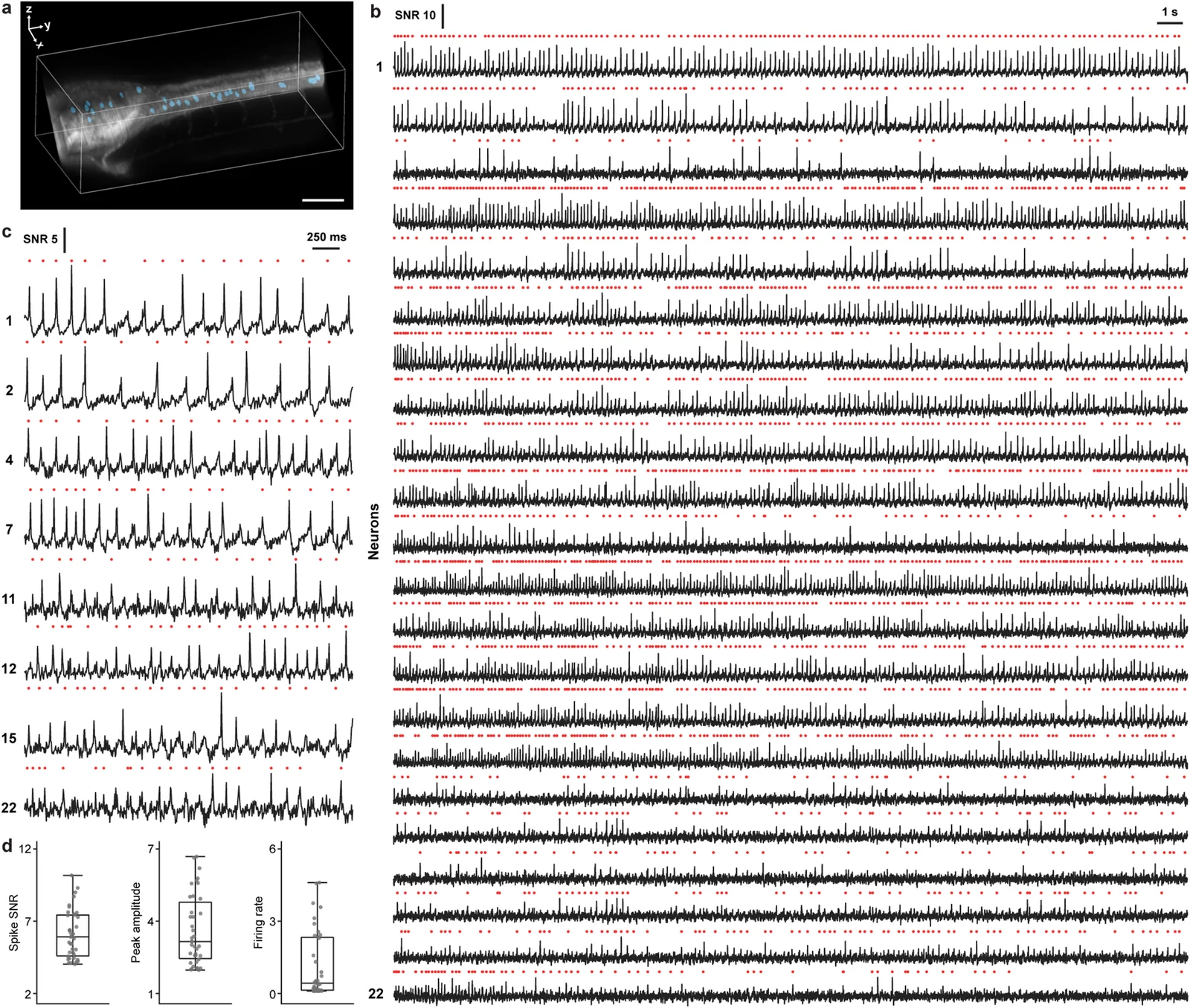
In vivo three-dimensional voltage imaging of neuronal activity in the larval zebrafish hindbrain and spinal cord. (**a**) Three-dimensional rendering of the reconstructed volumetric dataset. Segmented neurons are overlaid in blue. Scale bar, 100 μm. (**b**) Voltage traces extracted from the denoised imaging data using the neuronal ROIs marked in (**a**). Red dots indicate detected spike events. (**c**) Expanded views of representative voltage transients from selected neurons. Denoised traces correspond to the first 3 s of the recordings in (**b**). Red dots indicate detected spike events. (**d**) Spike signal-to-noise ratio (SNR), peak spike amplitude (% ΔF/F_o_), and firing rate (Hz) across segmented neurons (39 ROIs, from left to right). Box-and-whisker plots with individual values overlaid.

We next performed single-plane imaging of the larval zebrafish brain at 200 Hz (Fig. 3a). We observed voltage signals in neurons located in the habenula, cerebellum, and medulla oblongata (Fig. 3b). Extracted voltage signals from these neurons showed membrane potential fluctuations (Fig. 3c,d). We then performed three-dimensional voltage imaging of neuronal activity in the brain and spinal cord of live larval zebrafish at imaging rates of 200 VPS. Volumetric imaging was performed by sequentially scanning oblique image planes and reconstructing them into three-dimensional volumes using affine transformation. To maximize volumetric coverage while maintaining the same imaging configuration, the larval zebrafish were mounted at a tilted orientation aligned with the oblique light-sheet (Fig. 3e). This sample mounting strategy enabled expanded brain-wide coverage, allowing detection of spike-like voltage transients from neurons located in the olfactory epithelium, telencephalon, and medulla oblongata (Fig. 3f,g and Supplementary Video 4). To extract neuronal activity and detect spikes from the volumetric recordings containing a large number of neurons distributed across multiple brain regions, we utilized VolPy^45^, an automated analysis pipeline for voltage imaging data. Spike-like voltage transients were detected from neurons distributed across multiple brain regions (Fig. 3h,i). Using the same imaging configuration, we also recorded volumetric voltage activity across the hindbrain and spinal cord at 200 VPS under normal mounting conditions (Fig. 4a, Supplementary Video 5). Neuronal cell bodies were segmented from denoised volumes, and spike-like voltage transients were detected across neurons distributed throughout the hindbrain and spinal cord (Fig. 4b,c). We quantified spike SNR, peak spike amplitude, and firing rate across segmented neurons (Fig. 4d), confirming robust spiking activity throughout the hindbrain and spinal cord. These results demonstrated reliable membrane potential detection across both brain and spinal cord regions in the live larval zebrafish.

**Fig. 5:**
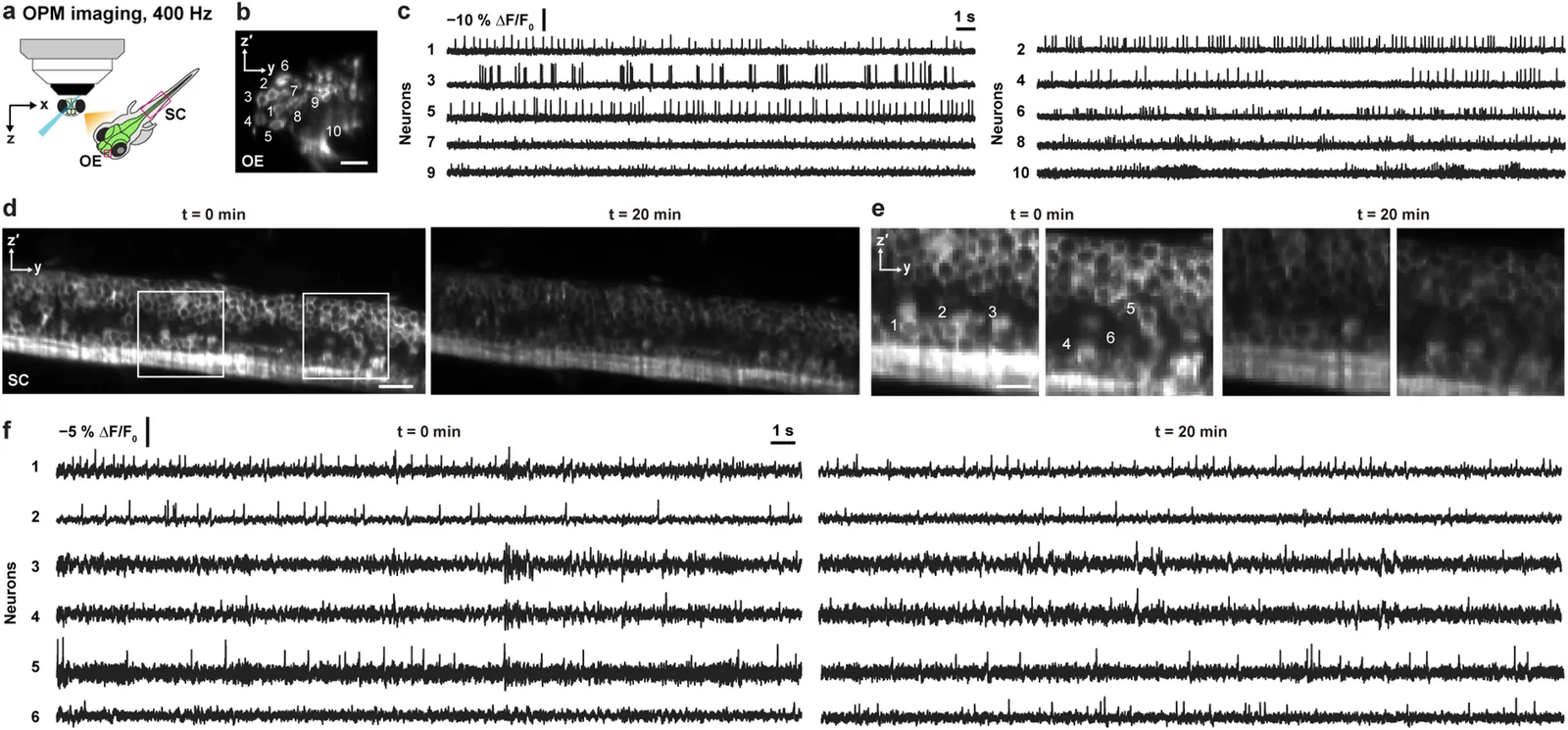
Long-term in vivo voltage imaging of neuronal activity in the larval zebrafish brain and spinal cord. (**a**) Schematic of the oblique plane microscopy (OPM) experimental setup. Red boxes indicate the targeted imaging regions in the larval zebrafish: olfactory epithelium (OE) and spinal cord (SC). (**b**) In vivo single-plane voltage imaging of the olfactory epithelium in larval zebrafish expressing ElectraOFF, acquired at 400 Hz. Scale bar, 20 μm. (**c**) Neuronal voltage traces extracted from the denoised imaging data using the neuronal ROIs marked in (**b**). (**d**) In vivo single-plane voltage imaging of the spinal cord in larval zebrafish expressing ElectraOFF, continuously recorded at 400 Hz for 20 min. Scale bar, 20 μm. (**e**) Magnified views of the boxed regions in (**d**). Scale bars, 10 μm. (**f**) Neuronal voltage traces extracted from the denoised imaging data using the neuronal ROIs marked in (**e**), comparing the neuronal activity during the initial 30 s and after 20 min of recording.

Despite these capabilities, long-duration recordings with Ace-mNeon2-Kv2.1 were limited by photobleaching (Supplementary Fig. 6). To address the photobleaching bottleneck, we performed long-duration voltage imaging using ElectraOFF, a recently developed eFRET-based GEVI derived from the photostable mBaoJin fluorescent protein, which demonstrates over 6-fold improved photostability compared to Ace-mNeon2 and enables long-term in vivo voltage recordings^46^. In our system, single-plane voltage imaging of the olfactory epithelium and spinal cord in transgenic larval zebrafish expressing ElectraOFF pan-neuronally was performed at 400 Hz (Fig. 5a). We observed spike-like voltage transients in neurons of the olfactory epithelium (Fig. 5b,c). We next performed continuous recordings of the spinal cord over 20 min, demonstrating that neuronal structures remained visible and fluorescence signals were maintained (Fig. 5d,e). Neuronal voltage signals remained detectable throughout 20 min of continuous recording (Fig. 5f). These results demonstrated the effectiveness of the photostable voltage indicator for long-duration in vivo voltage imaging in our OPM system.

## Discussion

Our study demonstrates three-dimensional voltage imaging of large neuronal populations in the live larval zebrafish using oblique plane microscopy and QFDBD–QUAS–driven expression of a fully genetically encoded voltage indicator. By leveraging high-speed oblique plane microscopy together with soma-enriched membrane targeting of Ace-mNeon2-Kv2.1, we achieved volumetric recordings across an extended field of view while preserving detectability of spike-like voltage transients and millisecond-scale temporal resolution. Volumetric recordings at up to 200 volumes per second, corresponding to a 5 ms sampling interval per volume, enabled observation of spike-like voltage transients in both brain and spinal cord, with kinetics characterized by an approximately 6 ms FWHM as measured in single-plane recordings at 1002 Hz.

Single-objective light-sheet microscopy approaches such as OPM have been recognized as promising platforms for high-speed volumetric imaging with simplified optical access, although their use for voltage imaging had not yet been demonstrated^47^. Our results demonstrate this capability, extending the applicability of single-objective light-sheet microscopy techniques^29–35^ from structural and organism-level physiological imaging to the direct measurement of rapid membrane potential dynamics underlying fast neuronal events. The fully genetically encoded indicator enables dye-free voltage imaging, eliminating the need for exogenous dye loading required in chemigenetic approaches. This genetic strategy provides stable soma-enriched membrane expression, simplifying experimental workflows and improving reproducibility across experiments.

Despite these advances, several limitations remain. First, while the system provides sufficient spatial resolution to resolve neuronal cell bodies and extract voltage signals, conventional OPM configurations are unable to use the full numerical aperture (NA) of the primary objective for fluorescence detection, resulting in reduced resolution and light collection efficiency^33^. The spatial resolution also varies across the imaging field of view, limiting the performance of the automated segmentation methods in the lower resolution regions. In addition, volumetric voltage imaging is constrained by the available photon budget. Although the camera is capable of operating at higher frame rates, the practical imaging speed is limited by signal-to-noise considerations. Increasing acquisition speed reduces photon counts per voxel, degrading SNR; therefore, an optimal volumetric rate should balance exposure time, photon collection, and temporal resolution. Scaling to larger fields of view further distributes finite photons across space and time, imposing inherent trade-offs among spatial resolution, temporal precision, and signal fidelity, making whole-brain imaging at comparable spatiotemporal resolution challenging. Compensating for reduced photon counts by increasing excitation laser power accelerates photobleaching, thereby limiting long-term recordings. Furthermore, the stripe artifacts arising from light-sheet illumination can obscure neuronal signals and introduce time-varying intensity fluctuations on extracted voltage traces, posing a challenge for reliable voltage detection in volumetric recordings. Moreover, high-speed volumetric acquisition generates large data volumes that limit recording duration due to memory bandwidth, data transfer, and storage constraints^21^. Addressing these optical, physical, and computational limitations will be essential for achieving brain-wide, long-duration, high-speed voltage imaging in future implementations.

Several opportunities remain for further development. Improved spatial resolution via optical advances such as the use of a custom tertiary objective^33^ would enable automated detection of neurons in densely labeled regions. Regarding molecular tools, the photostable voltage indicators effectively address the photobleaching bottleneck inherent to long-duration volumetric acquisition. Technical advances in high-speed data acquisition hardware, voltage imaging platforms for freely swimming larval zebrafish, and storage systems capable of handling the large data volumes generated by continuous volumetric recordings, combined with the molecular tool development, would broaden the scope of accessible biological investigations, including seizure-induced neuronal dynamics, stimulus-evoked activity patterns, and causal relationships between circuit-level voltage dynamics and behavior.

In summary, our study presents the effectiveness of oblique plane microscopy combined with fully genetically encoded voltage indicators for capturing neuronal activity in the live larval zebrafish. Our findings highlight the system’s capability to capture rapid neuronal events and observe fast voltage changes with high fidelity across distributed regions of the intact nervous system. This approach provides a practical platform for investigating neuronal circuit interactions in the zebrafish animal model, removing the experimental burden of exogenous dye handling.

## Methods

### Microscopy system description

The oblique plane microscope (OPM) was designed and optimized for high-speed volumetric voltage imaging. Optical simulations were performed using a commercial optical simulation software (Zemax OpticStudio, Ansys) to determine the optimal lens configuration and minimize optical aberrations across the target volumetric field of view. The optical components used in the Zemax simulation are summarized in Supplementary Table 1.

In the emission path shown in Fig. 1a, a primary objective (O1, f = 9 mm, XLUMPLFLN20XW, NA1.0, water, Olympus) was used to generate the oblique light-sheet and collect emitted fluorescence. The fluorescence light then passed through an achromatic lens (L1, f = 150 mm, 49–285, Edmund Optics), two Plössl lenses (L2, f = 75 mm, 2 × 49–285, Edmund Optics; L3, f = 100 mm, 2×AC508-200-A, Thorlabs), and another identical achromatic lens (L4, f = 150 mm, 49–285, Edmund Optics) to conjugate the pupil planes of O1 and a secondary objective (O2, f = 9 mm, UPLSAPO20X, NA0.75, Olympus). The intermediate image magnification (1.33) corresponded to the refractive index ratio between the immersion media of O1 (water, *n* = 1.33) and O2 (air, *n* = 1.00). A third objective lens (O3, f = 10 mm, 58–373, 20× NA0.60, Edmund Optics) was positioned at an angle of 38 degrees relative to O2. The intermediate image was directed into O3 and then focused through a camera tube lens (L5, f = 140 mm, Nikon) onto an intensified camera (HiCAM Fluo, Lambert Instruments). For an alternative detection configuration, a tube lens (L5, f = 100 mm, Nikon) was paired with an sCMOS camera (Zyla 5.5, Andor, Oxford Instruments). The effective pixel size at the sample plane was determined as the camera pixel size divided by the calculated system magnification. The effective pixel sizes were 0.53 μm (1.06 μm with 2 × 2 binning) for the intensified camera and 0.98 μm with 2 × 2 binning for the sCMOS camera.

For excitation, we used a 488 nm laser (Cobolt 06-MLD, HÜBNER Photonics) and a Powell lens (P1, fan angle = 30 degrees, 43–473, Edmund Optics) for a uniform light-sheet illumination. The diverging beam from the Powell lens was focused into a line after passing through a plano-convex cylindrical lens (C1, f = 50 mm, LJ1695RM-A, Thorlabs). Two identical achromatic lenses (R1 and R2, f = 100 mm, AC254-100-A, Thorlabs) for a 4f relay system and an adjustable slit was positioned between the relay lenses to modify the light-sheet’s numerical aperture (NA). The laser beam was focused onto a single-axis scanning galvo (GVS011/M, Thorlabs) after passing through the plano-convex cylindrical lens (C2, f = 75 mm, LJ1703RM-A, Thorlabs) and a dichroic mirror (DM1, FF495-Di03-25 × 36, Semrock). The galvo was controlled by an analog signal from a DAQ board (USB-6363, National Instruments), synchronized with the trigger output signals from the intensified camera. For image acquisition, custom software was implemented using MATLAB to control the galvo and DAQ board and commercial image acquisition software for the sCMOS camera (Solis, Andor, Oxford Instruments) and the intensified camera (Capture, Lambert Instruments) was used. All optical components used in the microscopy system are listed in Supplementary Table 2.

### Point spread function measurement

To characterize the resolution of our imaging system, the point spread function (PSF) was measured using 200 nm diameter yellow-green fluorescent beads (15700-10, Polysciences) embedded in 2% low melting-point agarose (Thermo Scientific). The measured 3-D PSF was fitted with a Gaussian function, and the full width at half maximum (FWHM) along the x, y, and z axes was calculated from the estimated standard deviation of the fitted Gaussian. The PSF measurements were performed for both the sCMOS camera and the intensified camera configurations. We note that the spatial resolution was degraded when using the intensified camera compared to the sCMOS camera (Supplementary Fig. 2). The spatial resolution degradation observed with the intensified camera was further confirmed using a wide-field microscope (Ts2, Nikon) equipped with a ×20 0.45 NA air objective (Supplementary Fig. 7).

### Zebrafish transgenesis

Transgenic zebrafish were generated using the Tol2 transposon system for the new transgenic *Tg(13xQUAS: Ace-mNeon2-Kv2.1)* and *Tg(13xQUAS: ElectraOFF)* lines. The coding sequences of Ace-mNeon2-Kv2.1 and ElectraOFF were cloned downstream of 13×QUAS regulatory elements to generate the *Tg(13xQUAS: Ace-mNeon2-Kv2.1)* and *Tg(13xQUAS: ElectraOFF)* constructs (Supplementary Figs. 8, 9). The Tg(HuC/elavl3:QFDBD-2xAD*-VP16*) driver line was obtained from the Fluorescent Reporter Zebrafish Cooperation Center (FRZCC 1027) and was not generated in this study. For transgenesis of *Tg(13xQUAS: Ace-mNeon2-Kv2.1)* and *Tg(13xQUAS: ElectraOFF)*, plasmid DNA (30 ng/µL) and Tol2 transposase mRNA (30 ng/µL) were microinjected into one-cell-stage embryos obtained from crosses between Tg(HuC/elavl3:QFDBD-2xAD*-VP16*) fish and nacre adults. Injected embryos were screened for fluorescence, and only fluorescence-positive larvae were raised to adulthood as F0 fish. F0 adults were crossed with nacre adults to generate F1 progeny, and again, only fluorescence-positive fish were selected and raised to adulthood. F1 adults were subsequently crossed with nacre adults to obtain F2 progeny. Fluorescence-positive F2 larvae at 4–6 dpf were used for imaging experiments.

### Zebrafish husbandry

All animal experiments involving zebrafish (*Danio rerio*) were conducted in accordance with the guidelines for animal research. All experimental protocols were approved by the Institutional Animal Care and Use Committee (IACUC) of KAIST (KA2025-032). All larvae were maintained on a 10-h dark, 14-h light cycle at 28 °C until at least 6 dpf. The sex of the larvae is not specified at this developmental stage and was therefore not determined.

### Sample preparation for imaging

For zebrafish experiments, transgenic larval zebrafish with a *casper* (*mitfa(w2/w2);mpv17(a9/a9)*) or *nacre* (*mitfa(w2/w2)*)^48^ mutant background were imaged at 4–6 dpf. To minimize motion artifacts, the larvae were paralyzed by bath incubation with 0.25 mg/ml of pancuronium bromide (Sigma-Aldrich) solution for 2–3 min, and paralysis was confirmed by the absence of movement in response to tactile stimulation. After paralysis, the larvae were exposed to 20 mM pentylenetetrazole (PTZ; Sigma-Aldrich)^22,49^ for 10 min to increase neuronal activity. A 100 mM PTZ stock solution was prepared in deionized water and diluted in standard fish water to a final concentration of 20 mM. Following PTZ exposure, the larval zebrafish were embedded in 2% low-melting-point agarose (Thermo Scientific) in a Petri dish. The agarose was allowed to solidify for 3–5 min to ensure stable mounting. After solidification, the dish was filled with 20 mM PTZ solution as the immersion medium during imaging.

### Confocal volumetric imaging of larval zebrafish brain

For volumetric structural imaging of larval zebrafish brain, the specimens were imaged using point-scanning confocal microscopy (C2 Plus, Nikon) equipped with a ×16 0.8 NA water dipping objective lens (CFI75 LWD 16XW, Nikon). Imaging was performed using a 488 nm excitation laser with a power of 0.45 mW. The images were acquired at a frame rate of 0.25 Hz with an image size of 2048 × 2048 pixels and a pixel size of 0.39 μm. Each volume consisted of 214 z-slices with a z-step size of 1.23 μm. The resulting volumetric field of view was 795.5 μm (*x*) × 795.5 μm (*y*) × 261 μm (*z*).

### In vivo voltage imaging of the zebrafish brain and spinal cord

In vivo voltage imaging was performed in transgenic larval zebrafish (4–6 dpf) expressing Ace-mNeon2-Kv2.1 under the QFDBD-QUAS system using a custom-built oblique plane microscope equipped with an sCMOS camera for single-plane imaging and an intensified camera for volumetric imaging. The sCMOS camera was operated in 16-bit mode, and the intensified camera in 12-bit mode. Both cameras were operated with 2 × 2 binning, corresponding to effective pixel sizes of 0.98 μm and 1.06 μm at the sample plane, respectively. The excitation laser (488 nm) was operated at 5 mW at the source for single-plane imaging and 50 mW at the source for volumetric imaging. For single-plane imaging, oblique images were acquired at 200 Hz for the zebrafish brain with an image size of 780 × 256 pixels and at 1002 Hz for the spinal cord with an image size of 780 × 100 pixels, with recording durations of up to 83 s. For long-duration voltage imaging, transgenic larval zebrafish expressing ElectraOFF pan-neuronally were imaged at 400 Hz, with an image size of 256 × 100 pixels and a recording duration of up to 20 min. For volumetric imaging, oblique images of 640 × 256 pixels were acquired at a camera frame rate of 5,600 Hz. During each galvo scanning cycle, 28 sequential images were captured, including 3 images discarded due to galvo settling, resulting in 25 x-slices per volume with a step size of up to 3.2–5.3 μm. The volumetric imaging rate was up to 200 volumes per second (VPS), determined by the camera frame rate and the number of oblique images per volume; at 200 VPS with 28 images per galvo scanning cycle (25 planes + 3 discarded), the camera operated at 5600 Hz, corresponding to a 5 ms temporal sampling interval per volume, with recording durations of up to 31 s due to data transfer bandwidth and memory storage constraints. Higher volumetric rates can be achieved by reducing the number of planes per volume, which decreases the field of view along the x-axis, or by increasing the step size between planes, which reduces axial spatial sampling. Sequential oblique image planes were reconstructed into three-dimensional volumes using affine transformation to correct for the oblique imaging geometry. The volumetric field of view was 127 μm (*x*) × 678 μm (*y*) × 217 μm (*z*). Imaging parameters for all datasets are summarized in Supplementary Table 3.

To determine the time window of increased neural activity following sample preparation, calcium imaging of the larval zebrafish brain expressing pan-neuronal cytosolic GCaMP was performed using the same preparation protocol described above, which showed increased neural activity approximately 10 min after the sample preparation, with a further increase observed at approximately 50 min into the recording (Supplementary Fig. 10). We note that 20 mM PTZ concentration has been used as a seizure model in the larval zebrafish^49^, and this concentration may be sufficient to induce seizure-like activity. Voltage imaging experiments were therefore conducted approximately 10 min after the sample preparation, prior to the time window of the further increase in neural activity.

### Data processing and analysis

For denoising voltage imaging data, we used SUPPORT (https://github.com/NICALab/SUPPORT), a self-supervised deep learning method that suppresses zero-mean noise by leveraging spatial and temporal dependencies among pixel values. SUPPORT models were trained separately for each dataset using 61-frame input patches with a batch size of 64, U-Net channel dimensions of 64, 128, 256, 512, and 1024, and a spatial blind-spot size of 1 (*t*) × 3 (*x*) × 3 (*y*). Spatial patch sizes of 61 (*t*) × 128 (*x*) × 128 (*y*) were used for training. Denoising was performed on a workstation equipped with dual Intel Xeon Gold 6226R CPUs, 128 GB RAM, and an NVIDIA GeForce RTX 4090 GPU. To assess the effectiveness of denoising on automated cell segmentation, Cellpose-SAM^50^ was applied to both raw and SUPPORT denoised data, demonstrating that denoising increased the number of segmented neuronal ROIs (Supplementary Fig. 4).

Regions of interest (ROIs) corresponding to neuronal cell bodies were manually drawn on temporally averaged projections of the denoised datasets using the ImageJ annotation tool, as automated detection was limited in densely labeled regions with overlapping neuronal structures. This average intensity projection was performed along the temporal axis and was used as a guide for the ROI mask definition. We used only manually defined ROI masks for neuronal structures with voltage signals, as only a limited number of the automatically segmented ROIs showed voltage signals within the recording time window (Supplementary Fig. 11). Voltage traces were extracted from denoised data within each manually defined ROI showing voltage signals and converted to fractional fluorescence change (ΔF/F_o_). Due to the negative-going response of the Ace-mNeon2 voltage indicator, action potentials appeared as transient decreases in fluorescence. For visualization in figures, traces were displayed as − ΔF/F_o_. Baseline fluorescence for ΔF/F_o_ calculation was estimated using a temporal moving average with a window length of 0.3 s, and the window size was adjusted according to the acquisition frame rate. To extract neuronal activity and detect spikes from the volumetric recordings, we utilized VolPy, an automated voltage imaging analysis pipeline, with manually defined ROI masks provided as input. SNR traces were calculated by dividing the background-removed fluorescence traces (detrended traces) by the estimated baseline noise. Spike detection and extraction were performed by identifying local maxima, utilizing either hard-thresholding of the − ΔF/F_o_ traces or an adaptive thresholding method applied to the SNR traces. Parameters were adjusted according to the characteristics of each dataset. Detected spikes were temporally aligned to their peak and averaged to compute representative spike waveforms. The full width at half maximum (FWHM) was calculated from the averaged waveform. The spike SNR was calculated for each ROI as the ratio of spike amplitude to the estimated noise level to quantify detectability of spike-like voltage transients. The peak spike amplitude was defined as the maximum value of the detected spike waveform, and firing rate was calculated by dividing the total number of detected spikes by the recording duration. Photobleaching was characterized by quantifying the gradual decrease in baseline fluorescence across the recording duration. The bleaching rate was reported as percentage change in ΔF/F_o_ per second (% ΔF/F_o_/s), calculated from each ROI (Supplementary Fig. 6).

## Supplementary Information

Below is the link to the electronic supplementary material.


Supplementary Material 1



Supplementary Material 2



Supplementary Material 3



Supplementary Material 4



Supplementary Material 5



Supplementary Material 6


## Data Availability

The datasets generated and/or analysed during the current study are available in the Zenodo repository (https://zenodo.org/records/19235146). The plasmid used for the generation of the ElectraOFF zebrafish line and its full sequence are available from the WeKwikGene repository (https://wekwikgene.wllsb.edu.cn/plasmids/0000566). The transgenic zebrafish lines generated in this study will be made available upon reasonable request to the corresponding authors.
