# Supplementary material for: Three-dimensional voltage imaging in live larval zebrafish brains using fully genetically encoded voltage indicator
Source: Sci Rep. 2026 Jun 16;16:27491. doi: 10.1038/s41598-026-58107-8 (PMC13534597; doi:10.1038/s41598-026-58107-8)
Supplement: Supplementary file 1 [file 41598_2026_58107_MOESM6_ESM.pdf]

Three-dimensional voltage imaging in live larval zebrafish brains using fully genetically encoded voltage indicator

SUPPLEMENTARY INFORMATION

Document S1. Supplementary Figures 1–11 and Supplementary Tables 1–3

Supplementary Video 1. Confocal z-stack imaging of the larval zebrafish brain.

Supplementary Video 2. Neuronal structural imaging in volumetric OPM.

Supplementary Video 3. In vivo voltage imaging of the larval zebrafish spinal cord at 1,002 Hz.

Supplementary Video 4. Volumetric OPM imaging of the larval zebrafish brain using tilted sample mounting.

Supplementary Video 5. Volumetric OPM imaging of the larval zebrafish hindbrain and spinal cord using conventional sample mounting.

SUPPLEMENTARY FIGURES

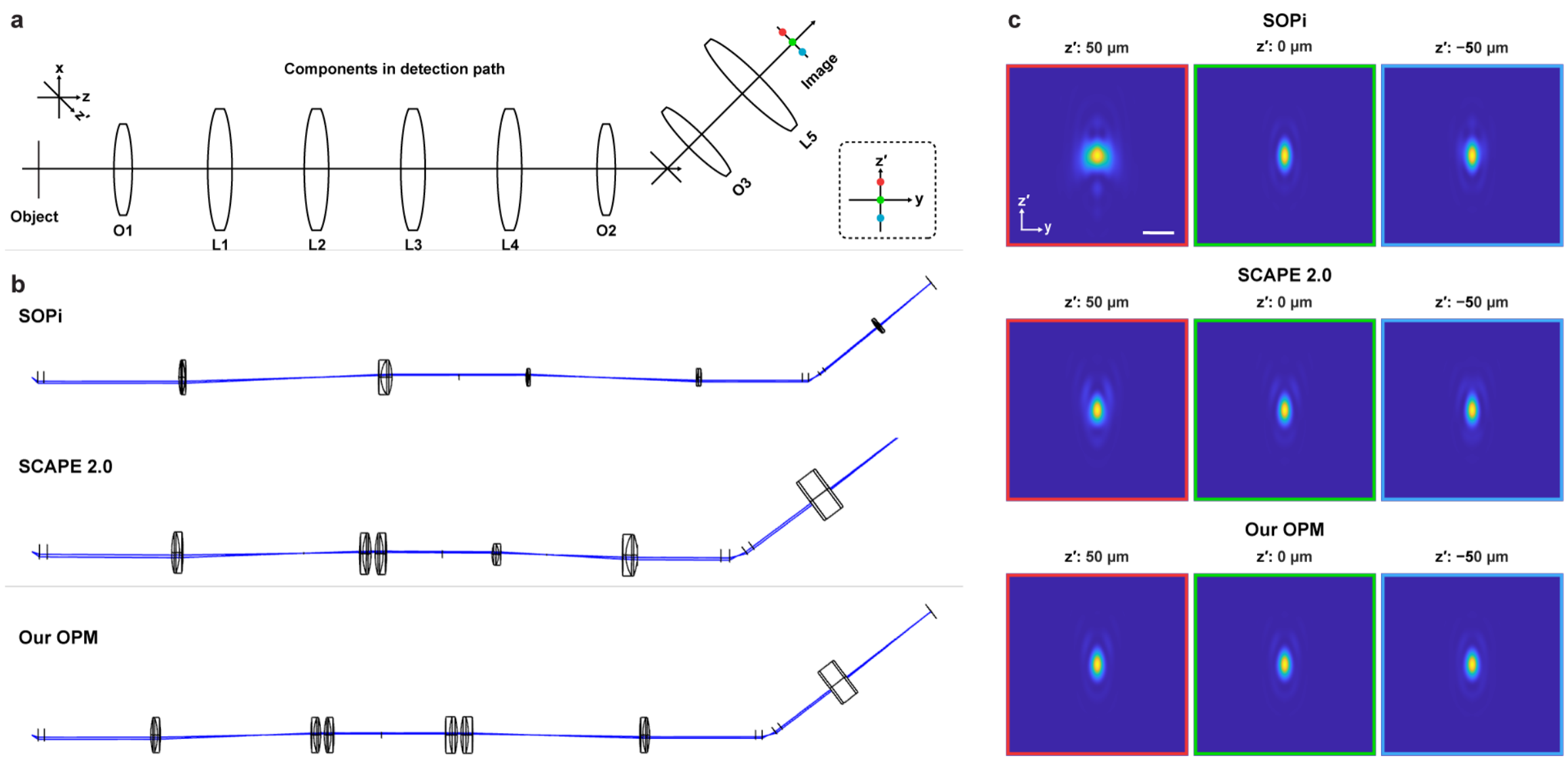

Supplementary Figure 1: Optical simulation of the oblique plane microscope.

a, Schematic of the optical components in the detection path, including the primary objective (O1), relay lenses (L1–L4), secondary objective (O2), third objective lens (O3), and camera tube lens (L5). The inset on the right illustrates the local coordinate system at the image plane, where the red, green, and blue dots represent the axial positions.

b, Optical models of the detection path implemented in the optical simulation software for SOPi (top), SCAPE 2.0 (middle), and our OPM (bottom).

c, Simulated point spread functions (PSFs) for SOPi (top), SCAPE 2.0 (middle), and our OPM (bottom) at varying depths. The red, green, and blue borders denote the corresponding axial planes marked by the colored dots in **a**. Scale bar, 1 µm.

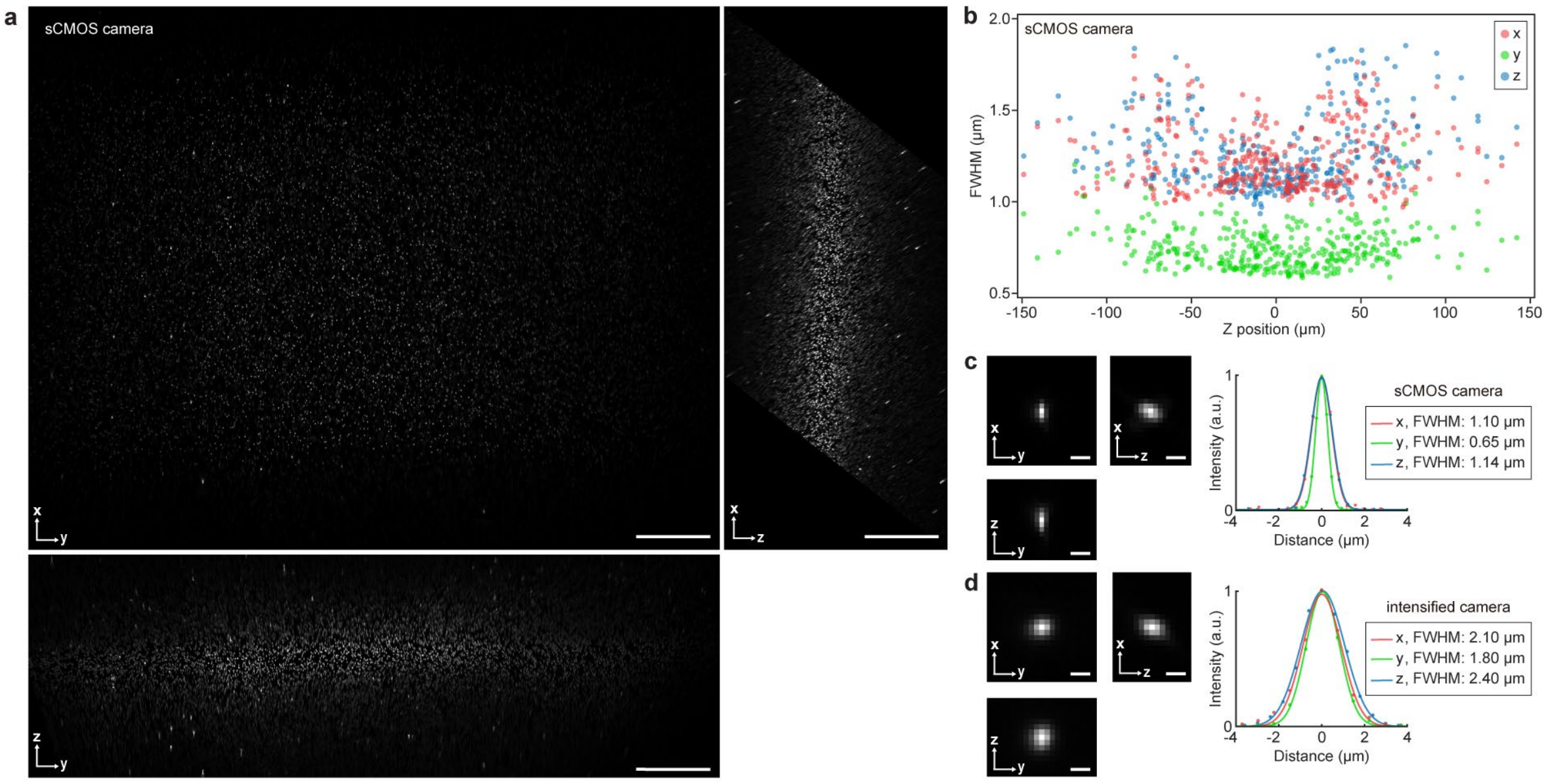

Supplementary Figure 2: Resolution characterization of OPM with sCMOS and intensified cameras.

a, Volumetric imaging of 200 nm diameter fluorescent beads embedded in agarose, acquired with the sCMOS camera. Maximum intensity projections along all three axes from skew-corrected raw data over a volume of 735 µm (*x*) × 936 µm (*y*) × 374 µm (*z*). Scale bars, 100 µm.

b, Spatial resolution measured as full width at half maximum (FWHM) of individual beads along the x (red), y (green), and z (blue) axes, acquired with the sCMOS camera.

c, Representative bead image at the center of the field of view, acquired with the sCMOS camera. Maximum intensity projections along all three axes from skew-corrected raw data. The FWHM values along the x, y, and z axes were approximately 1.10 µm, 0.65 µm, and 1.40 µm, respectively. Scale bars, 2 µm.

d, Representative bead image at the center of the field of view, acquired with the intensified camera. Maximum intensity projections along all three axes from skew-corrected raw data. The FWHM values along the x, y, and z axes were approximately 2.10 µm, 1.80 µm, and 2.40 µm, respectively. Scale bars, 2 µm.

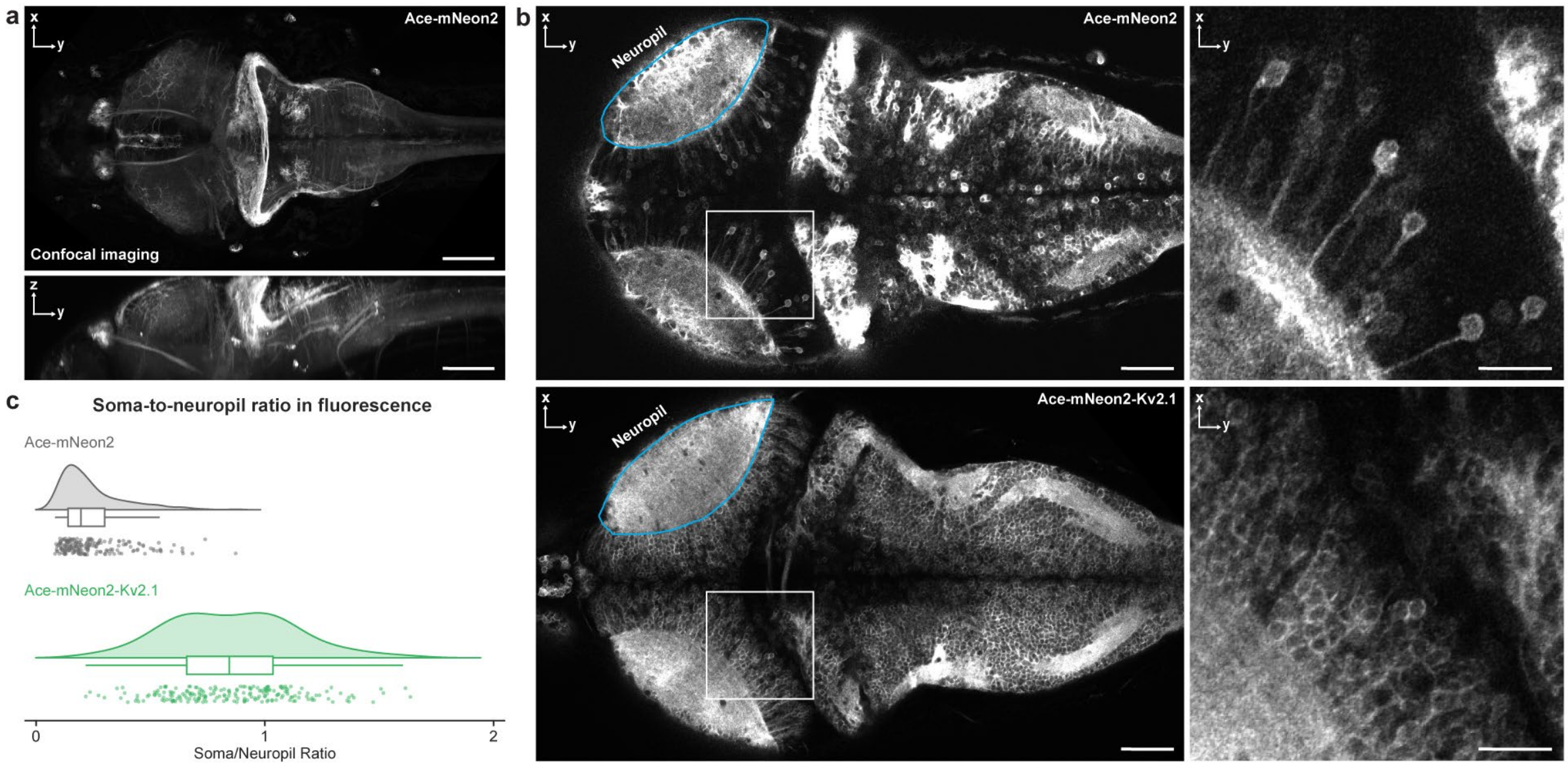

Supplementary Figure 3: Comparison of soma localization between Ace-mNeon2 and Ace-mNeon2-Kv2.1.

a, Volumetric imaging of the larval zebrafish brain pan-neuronally expressing Ace-mNeon2 using point-scanning confocal microscope. Maximum intensity projections along the axial and lateral directions. Scale bars, 100 µm.

b, Representative images (cropped field of view) at a selected imaging depth from the volumetric data shown in **a** (top, Ace-mNeon2) and **Fig. 1d** (bottom, Ace-mNeon2-Kv2.1), showing the optic tectum, cerebellum, and medulla oblongata. Images are displayed with adjusted contrast to enhance visibility. Manually segmented neuropil regions are overlaid in blue. Magnified views of the white boxed regions (optic tectum) are shown on the right. Scale bars, 50 µm (left) and 20 µm (right).

c, Soma-to-neuropil fluorescence ratio, calculated as the mean fluorescence intensity of soma ROIs divided by the mean fluorescence intensity of neuropil ROIs, for Ace-mNeon2 (gray) and Ace-mNeon2-Kv2.1 (green). The fluorescence ratio is displayed as a raincloud plot showing the data distribution, box-and-whisker plot, and individual data points. Each dot represents one neuronal ROI (200 ROIs).

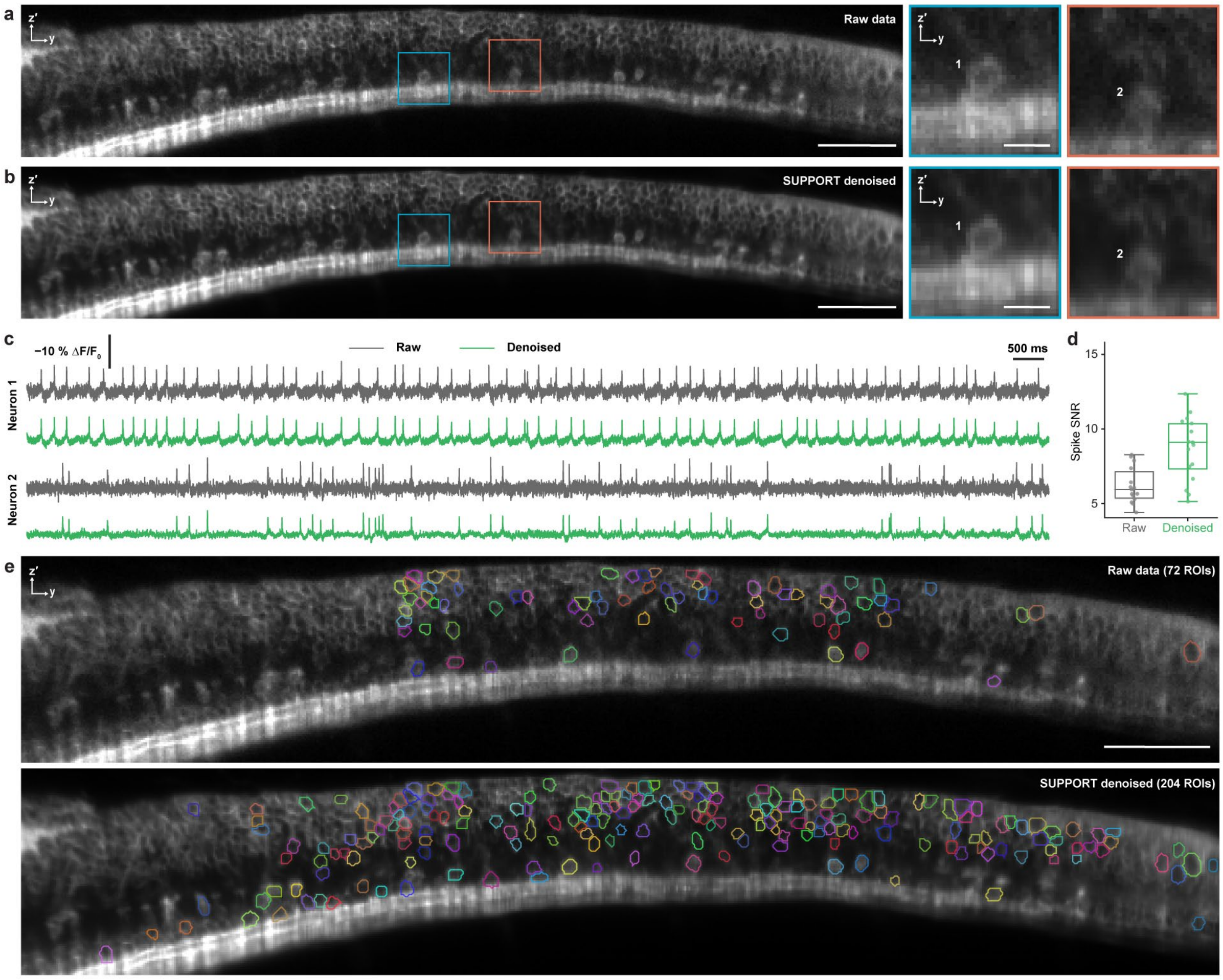

Supplementary Figure 4: Denoising of spinal cord voltage imaging data.

a, Representative single-frame image of the larval zebrafish spinal cord acquired at 1,002 Hz (raw data). Magnified views of the blue and orange boxed regions are shown on the right. Scale bars, 50 µm (left) and 10 µm (right).

b, Representative single-frame image after SUPPORT denoising. Magnified views of the blue and orange boxed regions are shown on the right. Scale bars, 50 µm (left) and 10 µm (right).

c, Voltage traces extracted from raw data (gray) and SUPPORT denoised data (green) using the neuronal ROIs shown in **a** and **b**, respectively.

d, Spike signal-to-noise ratio (SNR) of raw data (gray) and SUPPORT denoised data (green) across the neuronal ROIs (17 ROIs). Box-and-whisker plot with individual values overlaid.

e, Neuronal ROIs automatically segmented by Cellpose-SAM from raw data (top; 72 ROIs) and SUPPORT denoised data (bottom; 204 ROIs). Scale bar, 50 µm.

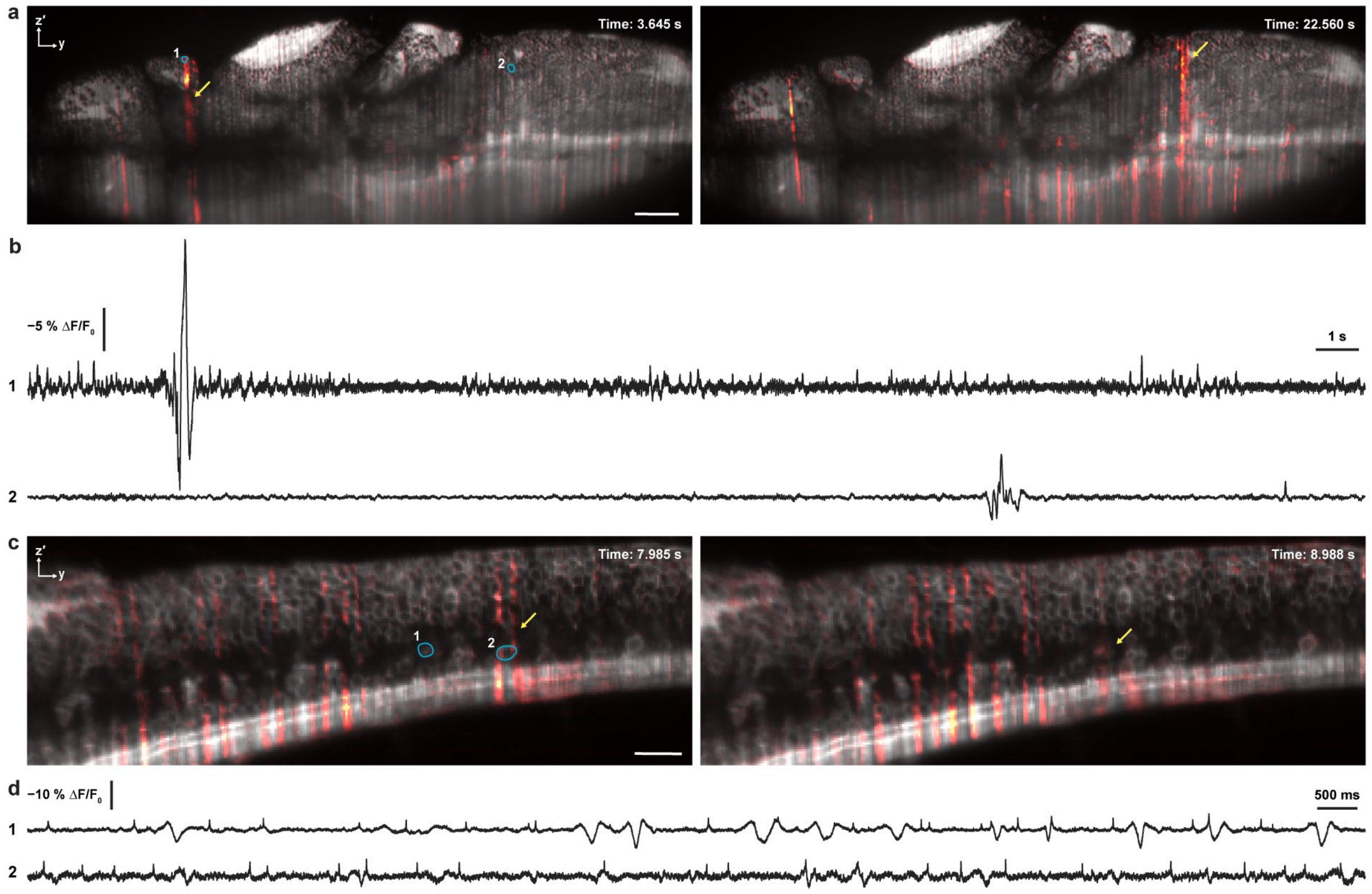

Supplementary Figure 5: Stripe artifacts in brain and spinal cord voltage imaging data.

a, Representative frames from the denoised imaging data of brain voltage imaging showing stripe artifacts. The baseline fluorescence (gray) and activity component (hot colormap) are overlaid. Yellow arrows indicate representative stripe artifacts. Manually segmented neuronal regions of interest (ROIs) are overlaid in blue. Scale bar, 50 µm.

b, Voltage traces extracted from segmented neurons, showing stripe artifacts as large-amplitude deflections that transiently obscure neuronal signals.

c, Representative frames from the denoised imaging data of spinal cord voltage imaging showing stripe artifacts. The baseline fluorescence (gray) and activity component (hot colormap) are overlaid. Yellow arrows indicate representative stripe artifacts. Manually segmented neuronal regions of interest (ROIs) are overlaid in blue. Scale bar, 20 µm.

d, Voltage traces extracted from segmented neurons, showing stripe artifacts as large-amplitude deflections that transiently obscure neuronal signals.

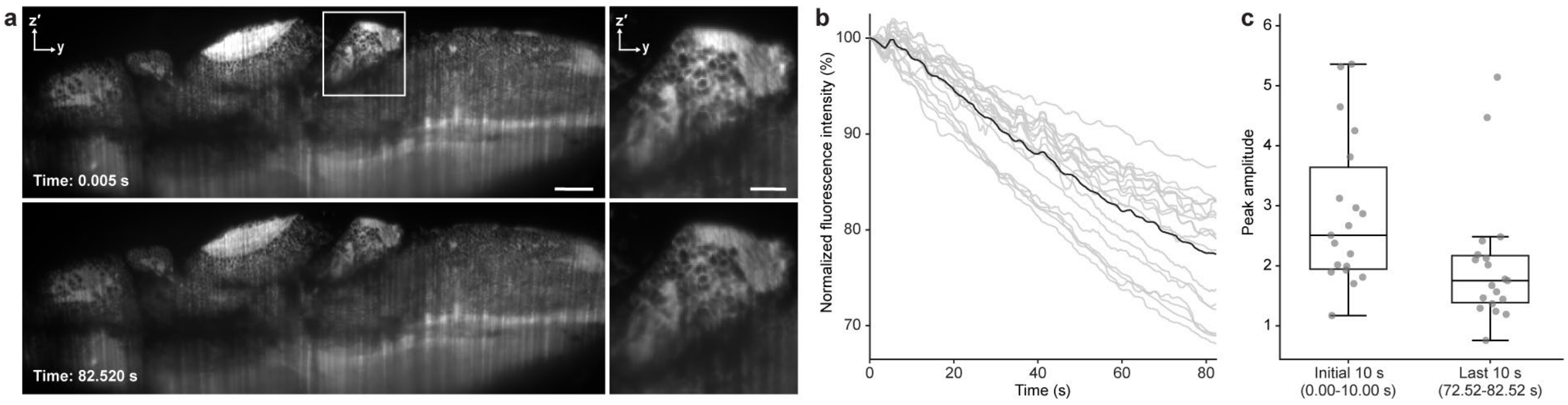

Supplementary Figure 6: Photobleaching during voltage imaging recording.

a, Representative OPM images of the larval zebrafish brain at the initial (t = 0.005 s, top) and last (t = 82.520 s, bottom) time points of the recording. Magnified views of the white boxed regions are shown on the right. Scale bars, 50 µm (left) and 20 µm (right).

b, Normalized fluorescence intensity over time for individual neuronal ROIs (gray) and the mean across all ROIs (black), showing the photobleaching profile (19 ROIs; averaged bleaching rate: 0.272% $\Delta F/F_0$/s), corresponding to a total fluorescence drop of approximately 20% over 83 s recording.

c, Peak spike amplitude (% $\Delta F/F_0$) during the initial 10 s window (0.00–10.00 s) and the last 10 s window (72.52–82.52 s) across segmented neurons (19 ROIs). Peak spike amplitude decreased by approximately 19% between the initial and last 10 s windows. Box-and-whisker plots with individual values overlaid.

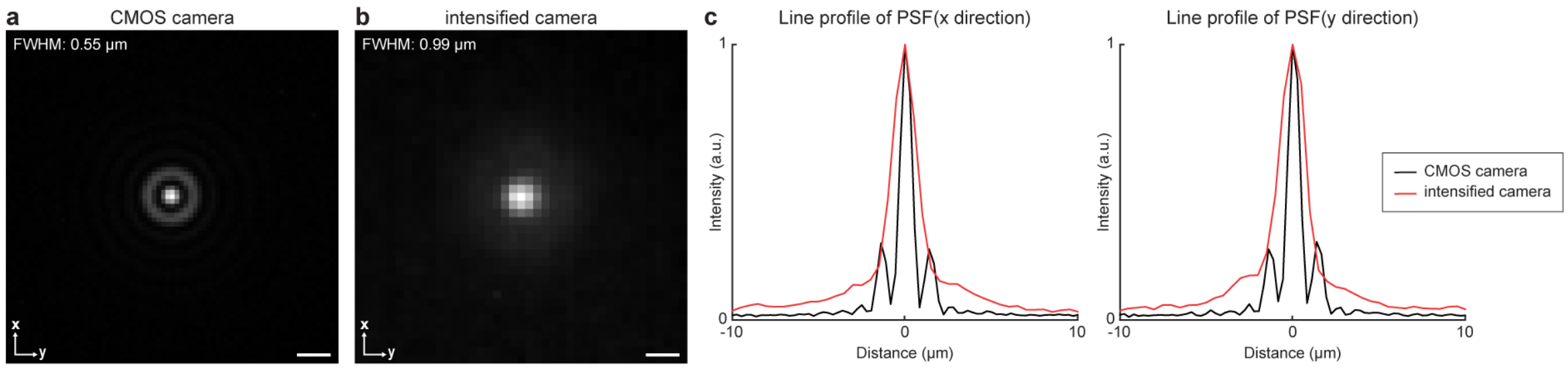

Supplementary Figure 7: Comparison of spatial resolution between the CMOS and intensified cameras.

a, Representative point spread function (PSF) image of a 200 nm fluorescent bead on a glass slide, acquired using a wide-field microscope (Ts2, Nikon) equipped with a ×20, 0.45 NA air objective and a CMOS camera (acA1920-155um, Basler). The measured full width at half maximum (FWHM) was 0.55 µm. Scale bars, 2 µm.

b, Representative PSF image of the 200 nm fluorescent bead, acquired using the same wide-field microscope equipped with an intensified camera. The measured FWHM was 0.99 µm. Scale bars, 2 µm.

c, Cross-sectional line profiles of the PSFs along the x (left) and y (right) directions for the CMOS camera (black) and the intensified camera (red), corresponding to the beads shown in **a** and **b**, respectively.

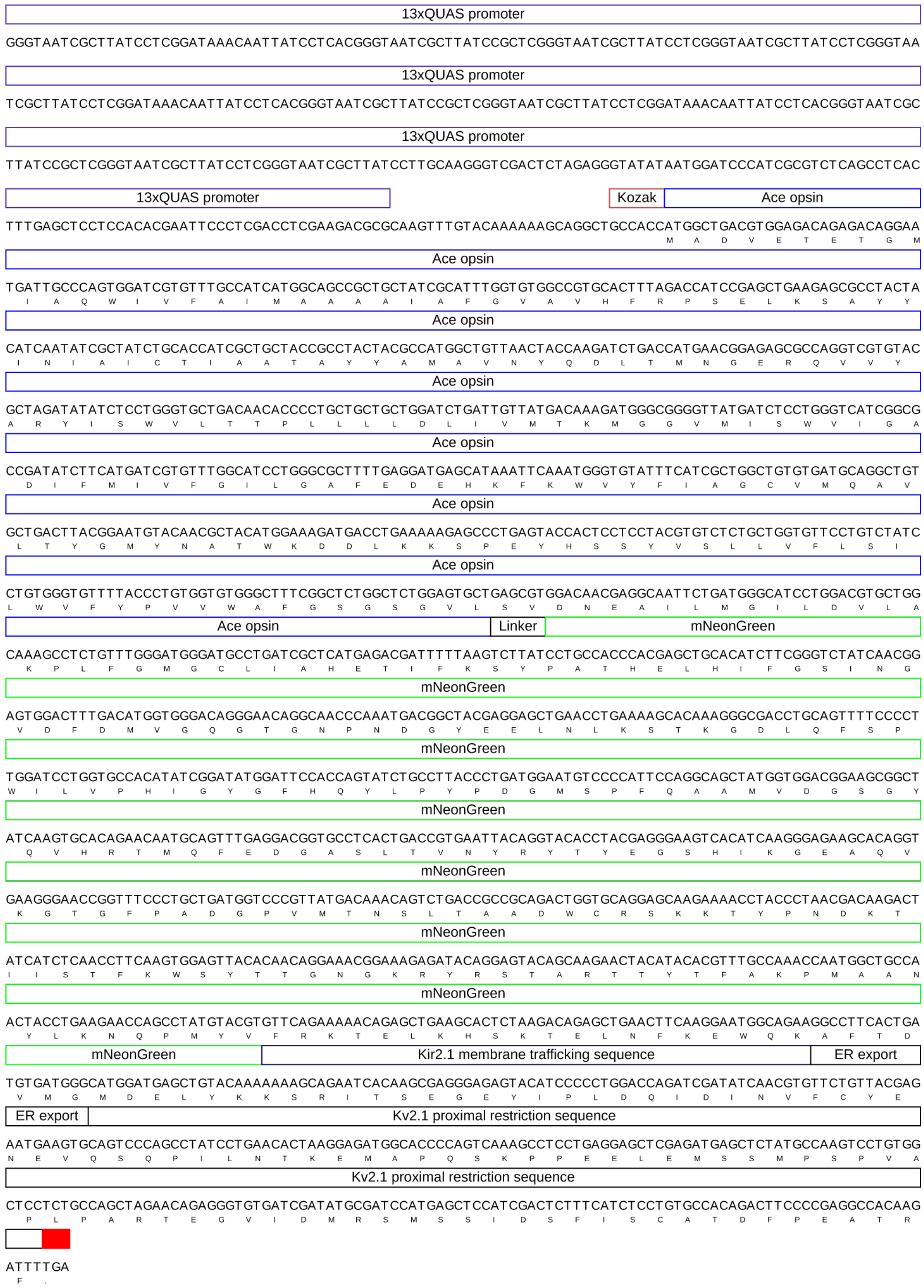

Supplementary Figure 8: DNA and amino acid sequences of 13xQUAS-Ace-mNeon2-Kv2.1.

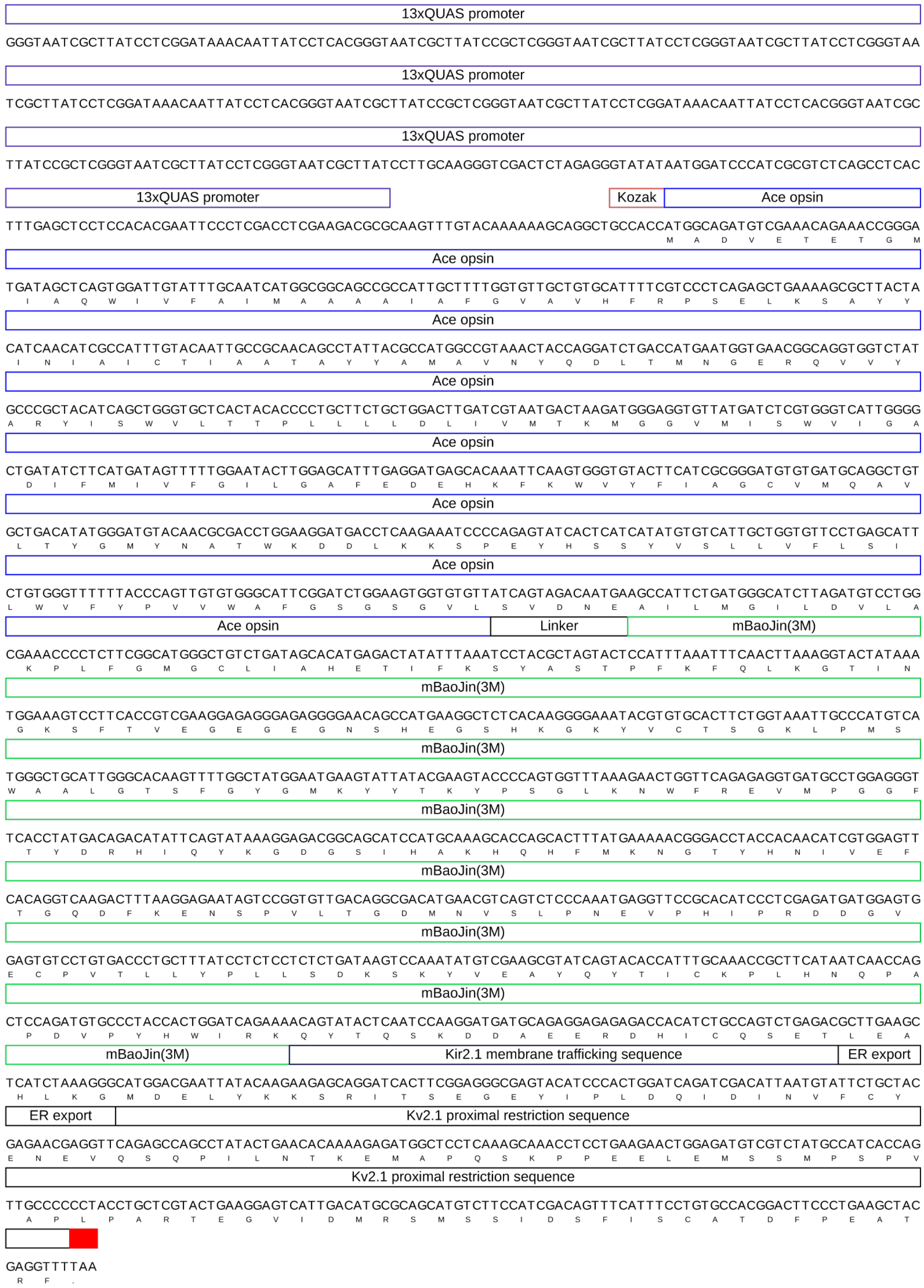

Supplementary Figure 9: DNA and amino acid sequences of 13xQUAS-ElectraOFF.

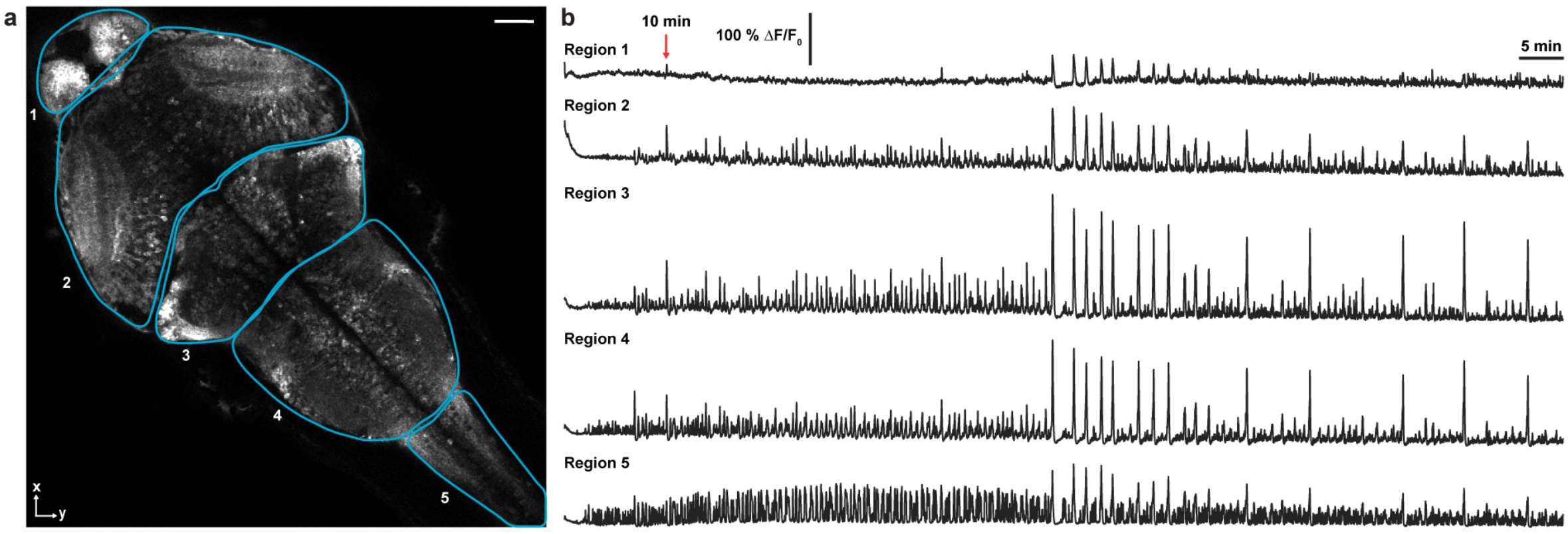

Supplementary Figure 10: Calcium imaging of increased neural activity in PTZ-exposed larval zebrafish.

a, Calcium imaging of the larval zebrafish brain expressing pan-neuronal cytosolic GCaMP7a [*Tg(HuC:Gal4); Tg(UAS:GCaMP7a)*] at 1 Hz using a point-scanning confocal microscope. Temporal maximum intensity projection of the calcium imaging dataset with manually segmented regions overlaid in blue (1–5), corresponding to the habenula (1), optic tectum (2), cerebellum (3), medulla oblongata (4), and spinal cord (5). Scale bar, 50 µm.

b, Calcium traces (% $\Delta F/F_0$) extracted from the regions indicated in **a** (top to bottom: habenula, optic tectum, cerebellum, medulla oblongata, and spinal cord). The red arrow indicates 10 min into the recording.

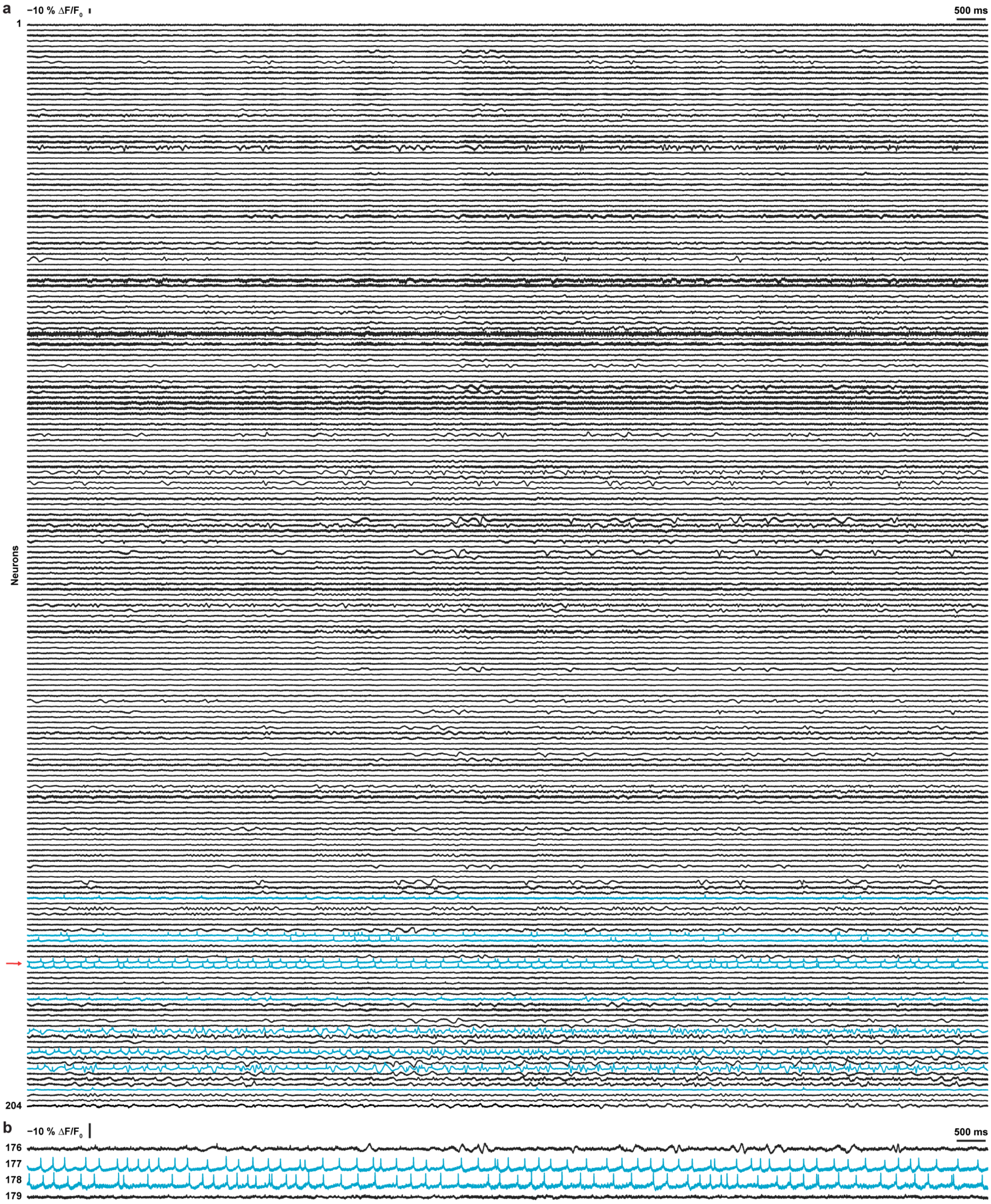

Supplementary Figure 11: Fluorescence traces from automatically segmented neuronal ROIs.

a, Fluorescence traces extracted from the denoised imaging data using the 204 neuronal ROIs automatically segmented by Cellpose-SAM, as shown in **Supplementary Fig. 4e**. The traces with voltage signals are highlighted in blue.

b, Expanded traces from ROIs indicated by the red arrow in **a**.

SUPPLEMENTARY TABLES

Table S1: Optical components used in the Zemax simulation.

	Optical components used for Zemax simulation (Part number, vendor)		
**Component**	**SOPi**	**SCAPE 2.0**	**Our OPM**
**O1**	20× NA 1.0 (Paraxial lens)	20× NA 1.0 (Paraxial lens)	20× NA 1.0 (Paraxial lens)
**O2**	20× NA 0.75 (Paraxial lens)	20× NA 0.75 (Paraxial lens)	20× NA 0.75 (Paraxial lens)
**O3**	20× NA 0.45 (Paraxial lens)	20× NA 0.6 (Paraxial lens)	20× NA 0.6 (Paraxial lens)
**L1**	f=200mm achromatic lens (AC508-200-A, Thorlabs)	f=150mm achromatic lens (49-285, Edmund Optics)	f=150mm achromatic lens (49-285, Edmund Optics)
**L2**	f=100mm achromatic lens (AC508-100-A, Thorlabs)	f=75mm Plössl lens (2×49-285, Edmund Optics)	f=75mm Plössl lens (2×49-285, Edmund Optics)
**L3**	f=100mm achromatic lens (AC254-100-A, Thorlabs)	f=60mm achromatic lens (AC254-60-A, Thorlabs)	f=100mm Plössl lens (2×AC508-200-A, Thorlabs)
**L4**	f=150mm achromatic lens (AC254-150-A, Thorlabs)	f=100mm achromatic lens (49-284, Edmund Optics)	f=150mm achromatic lens (49-285, Edmund Optics)
**L5**	f=100mm achromatic lens (AC254-100-A, Thorlabs)	f=200mm tube lens (TTL200-A, Thorlabs)	f=200mm tube lens (TTL200-A, Thorlabs)

Table S2: Optical components used in the oblique plane microscope system.

Component	Description	Part number (Vendor)
**O1**	20× NA 1.0, water	XLUMPLFLN20XW (Olympus)
**O2**	20× NA 0.75, air	UPLSAPO20X (Olympus)
**O3**	20× NA 0.6, air	58-373 (Edmund Optics)
**L1**	f=150mm achromatic lens	49-285 (Edmund Optics)
**L2**	f=75mm Plössl lens	2×49-285 (Edmund Optics)
**L3**	f=100mm Plössl lens	2×AC508-200-A (Thorlabs)
**L4**	f=150mm achromatic lens	49-285 (Edmund Optics)
**L5**	f=100mm tube lens	MXA20714 (Nikon)
	f=140mm tube lens	MXK60147 (Nikon) + MQD42075 (Nikon)
**Laser**	488 nm laser	Cobolt 06-MLD (HÜBNER Photonics)
**P1**	fan angle=30 degrees Powell lens	43-473 (Edmund Optics)
**C1**	f=50 mm plano-convex cylindrical lens	LJ1695RM-A (Thorlabs)
**C2**	f=75 mm plano-convex cylindrical lens	LJ1703RM-A (Thorlabs)
**R1**	f=100mm achromatic lens	AC254-100-A (Thorlabs)
**R2**	f=100mm achromatic lens	AC254-100-A (Thorlabs)
**Slit**	Adjustable slit	VA100CP (Thorlabs)
**Galvo**	single-axis large beam diameter galvo mirror	GVS011/M (Thorlabs)
**DM1**	dichroic mirror	FF495-Di03-25×36 (Semrock)
**F1**	emission filter	FF01-496/LP-25 (Semrock)
**DAQ**	DAQ board for analog input/output	USB-6363 (National Instruments)
**Camera**	sCMOS camera	Zyla 5.5 sCMOS (Andor, Oxford Instruments)
	intensified camera	HiCAM Fluo (Lambert Instruments)

Table S3: Imaging parameters for single-plane and volumetric voltage imaging.

Parameter	Single-plane (brain)	Single-plane (spinal cord)	Volumetric (200 VPS)	Long-duration
**GEVI**	Ace-mNeon2-Kv2.1	Ace-mNeon2-Kv2.1	Ace-mNeon2-Kv2.1	ElectraOFF
**Camera**	sCMOS	sCMOS	Intensified	sCMOS
**Bit depth**	16-bit	16-bit	12-bit	16-bit
**Binning**	2 × 2	2 × 2	2 × 2	2 × 2
**Camera frame rate**	200 Hz	1,002 Hz	5,600 Hz	400 Hz
**Image size (pixels)**	780 × 256	780 × 100	640 × 256	256 × 100
**Planes per volume**	—	—	25	—
**Effective pixel size**	0.98 µm	0.98 µm	1.06 µm	0.98 µm
**Axial step size (Δx)**	—	—	3.2–5.3 µm	—
**Field of view**	763 (y) × 250 (z') µm^2	763 (y) × 98 (z') µm^2	up to 127 (x) × 678 (y) × 217 (z) µm^3	250 (y) × 98 (z') µm^2
**Laser power (at source)**	5 mW	5 mW	50 mW	5 mW
**Laser power (at sample)**	0.45 mW	0.45 mW	5.29 mW	0.45 mW
**Excitation wavelength**	488 nm	488 nm	488 nm	488 nm
**Recording duration**	up to 83 s	up to 83 s	up to 31 s	up to 20 min
